# Neurocognitive subprocesses of working memory performance

**DOI:** 10.3758/s13415-021-00924-7

**Published:** 2021-06-21

**Authors:** Agatha Lenartowicz, Holly Truong, Kristen D. Enriquez, Julia Webster, Jean-Baptiste Pochon, Jesse Rissman, Carrie E. Bearden, Sandra K. Loo, Robert M. Bilder

**Affiliations:** 1 University of California Los Angeles, Los Angeles, CA, USA

**Keywords:** Working memory, EEG, Alpha, Theta, Gamma, P3, Cross-frequency coupling, Maintenance, Updating, Goal maintenance, RDoC

## Abstract

Working memory (WM) has been defined as the active maintenance and flexible updating of goal-relevant information in a form that has limited capacity and resists interference. Complex measures of WM recruit multiple subprocesses, making it difficult to isolate specific contributions of putatively independent subsystems. The present study was designed to determine whether neurophysiological indicators of proposed subprocesses of WM predict WM performance. We recruited 200 individuals defined by care-seeking status and measured neural responses using electroencephalography (EEG), while participants performed four WM tasks. We extracted spectral and time-domain EEG features from each task to quantify each of the hypothesized WM subprocesses: *maintenance (storage of content), goal maintenance*, and *updating*. We then used EEG measures of each subprocess as predictors of task performance to evaluate their contribution to WM. Significant predictors of WM capacity included contralateral delay activity and frontal theta, features typically associated with *maintenance (storage of content)* processes. In contrast, significant predictors of reaction time and its variability included contingent negative variation and the P3b, features typically associated with *goal maintenance* and *updating*. Broadly, these results suggest two principal dimensions that contribute to WM performance, tonic processes during maintenance contributing to capacity, and phasic processes during stimulus processing that contribute to response speed and variability. The analyses additionally highlight that reliability of features across tasks was greater (and comparable to that of WM performance) for features associated with stimulus processing (P3b and alpha), than with maintenance (gamma, theta and cross-frequency coupling).

## Introduction

Working memory (WM) was described decades ago as the cognitive ability to store information briefly online after it is no longer available to our senses (Baddeley, 1986). A workgroup convened by NIMH subsequently defined WM as *“…the active maintenance and flexible updating of goal/task relevant information (items, goals, strategies, etc.) in a form that has limited capacity and resists interference. These representations: may involve flexible binding of representations; may be characterized by the absence of external support for the internally maintained representations; and are frequently temporary, though this may be due to ongoing interference*” (NIMH, 2011). The importance of WM in cognitive neuroscience research is underscored by its extensive theoretical consideration in cognitive psychology (Baddeley, 2002; Cowan, 2008; Miller, 1956; Oberauer, Süß, Schulze, Wilhelm, & Wittmann, 2000), a multitude of validated assays (Luck & Vogel, 1997; Nee, Wager, & Jonides, 2007; Vogel & Machizawa, 2004), and an association with putative biological mechanisms (D’Esposito et al., 1998; Fuster & Alexander, 1971; Goldman-Rakic, 1995) that have suggested clear links between cognitive and neural function. WM impairments may comprise the most clinically salient cognitive disorders, having been reported across nearly all neurological conditions, including traumatic brain injury (Alexander, Stuss, Picton, Shallice, & Gillingham, 2007; Stuss & Benson, 1984) and neuropsychiatric disorders, including anxiety (Brewin, Gregory, Lipton, & Burgess, 2010), depression (Joormann & Gotlib, 2008), substance abuse (Bechara & Martin, 2004), attention deficit hyperactivity disorder (ADHD) (Loo et al., 2007), schizophrenia (Gold et al., 2010), and bipolar disorder (Bearden, Hoffman, & Cannon, 2001).

Despite the salience of WM in the neurocognitive assessment of neuropsychiatric syndromes, it has remained a challenge to understand how the putative neural mechanisms of WM contribute to WM performance and its impairments. Namely, WM is a complex behavioral construct with multiple component processes that can be impaired through different functional neuroanatomic pathways, and each of these may contribute uniquely to poor WM performance. For instance, the putative component function “maintenance” can involve short-term storage associated with local cortical activities (Compte, Brunel, Goldman-Rakic, & Wang, 2000; Lisman & Idiart, 1995), long-term memory functions that may recruit cortico-hippocampal interactions (Axmacher et al., 2007), and/or maintenance of the behavioral goals guiding the behavior that may rely on the fronto-parietal network (O’Reilly & Frank, 2006). The extent to which these diverse processes are involved in WM remains a topic of debate and likely varies markedly across different tasks (Cowan, 2008; Nee & Jonides, 2011). In addition, effective maintenance relies on effective encoding and its relation to contexts (*updating*); it requires interactions of maintenance processes with sensory and motor circuit functions, and/or with more complex semantic functions that engage extensive cortico-cortical networks. Thus, impairments in WM performance may stem from such interactions rather than from maintenance processes per se. For example, recent studies of ADHD report that visual attention processes during encoding predict neural responses during WM maintenance and other aspects of task performance, suggesting that attentional interactions during updating, rather than maintenance processes, may account for a portion of WM performance impairments in this population (Lenartowicz et al., 2014; Lenartowicz, Mazaheri, Jensen, & Loo, 2018). In contrast, in schizophrenia, the contribution of goal maintenance processes to deficits of WM have been well-documented, hypothesized to result from dysfunction of frontoparietal connectivity or corticostriatopallidothalamic circuits (Barch & Dowd, 2010). These observations highlight a need to understand the differential contributions of the functional anatomic bases of WM subprocess to clinically important disorders of brain and behavior.

The present study aimed to contribute to this understanding. Motivated by the NIMH Research Domain Criteria (RDoC) initiative (RDoC, Insel et al., 2010), we defined putative neural circuits considered important for WM, identified EEG indicators theoretically relevant to these circuits, and assessed their associations with WM behavioral measures in a heterogenous sample of individuals ascertained to capture clinically relevant variability in WM function. We did this in two steps. First, as noted above, we defined neural circuits putatively underlying WM: local-cortical circuits associated with *short-term memory storage* (Compte et al., 2000; Lisman & Idiart, 1995); cortico-hippocampal circuits necessary for WM operations that exceed span and enable *long-term memory storage* (Axmacher et al., 2007); and frontoparietal corticocortical circuits associated with *goal maintenance* and interference control (O’Reilly & Frank, 2006). WM “*updating*” is another important aspect of WM that we conceptualized as the phasic alteration of local circuits via subcortical-cortical circuits (D’Ardenne et al., 2012), occurring during stimulus processing and response selection. To provide neurophysiological indicators of each subprocess, we used electroencephalographic (EEG) metrics, previously associated with the putative neural circuits underlying the proposed neurocognitive constructs, during four different WM tasks, and then used dimension-reduction methods to construct simpler neurophysiological indicators of each circuit. The EEG signals include frontal gamma-range power, contralateral delay activity (CDA) and N170 potentials for *short-term storage*; frontal theta-range power, and gamma cross-frequency coupling (CFC) measures for *long-term storage*; alpha-range CFC and contingent negative variation (CNV) event-related potential (ERP) for *goal maintenance*; P3b ERP, and alpha-range power for *updating*. Some of these EEG measures were *a priori* linked to specific WM constructs (e.g., the CNV has long been seen as an EEG indicator of maintenance operations, and CDA during the lateralized change detection task has been validated as an index of WM capacity), but the interpretation of other EEG signals may depend on the task context within which these are collected. All measures, and the rationale for their selection, are described in detail in the Methods section. To test the validity of these derived measures, we regressed WM performance on the EEG circuit indicators. In sum, we examine the contribution of proposed WM functional circuits to WM performance, and do so both across WM tasks and in a clinically heterogeneous population.

## Methods and Materials

### Participants

We recruited 200 adults from the community to participate in the project: *“Multi-Level Assays of Working Memory and Psychopathology”*—supported by the National Institute of Mental Health (NIMH) Research Domains Criteria (RDoC) Initiative (PI: Bilder, R01-MH101478). To overcome biases associated with traditional diagnostic inclusion/exclusion criteria, the project studied adults who were care-seeking (CS, n = 150) and non-care-seeking (NCS, n = 50), and prioritized enrollment of patients with more severe symptoms to ensure a broad range of psychopathology and high variance in major symptom dimensions. The CS group comprised individuals who were seeking treatment for mental or emotional problems and responded to advertisements or were referred to the UCLA Neuropsychiatric Behavioral Health Service. The NCS group comprised individuals who responded to advertisements and had not sought behavioral health or substance-abuse services within 12 months preceding enrollment.

Additionally, participants satisfied the following inclusion criteria: aged 21–40 years; completed at least 8 years of formal education; general mental status, hearing, motor coordination, and cooperation sufficient to complete procedures; language proficiency in English; IQ estimate >70 (on WAIS-IV Vocabulary and Matrix Reasoning); visual acuity 20:50 or better within each eye (right and left eyes tested separately); no medical or neurological illness or treatment expected to have cognitive effects; no psychotropic or sedating drugs within 24 hours of exam; no long-acting antipsychotics or electroconvulsive therapy within the preceding 6 months; no substance abuse disorder diagnosis other than caffeine or nicotine in preceding 6 months; negative urinalysis results for THC, cocaine, amphetamine, opiates, and benzodiazepines; no contraindications to MRI exam (metal in the body, claustrophobia, or for females, pregnancy).

All participants underwent comprehensive diagnostic and neurocognitive assessments, the results of which have been presented elsewhere (pertinent details are provided in Supplemental Materials). In this report, we consider only a subset that are most directly relevant to the EEG features examined: overall disability as indexed by the World Health Organization Disability Affective Schedule (WHODAS 2.0), 32-item summary score, as well as indicators of anxiety and depression (Brief Psychiatric Rating Scale Anxiety and Depression [BRIEF] subscales; Patient-Reported Outcomes Information System [PROMIS] anxiety and depression sub-scales), known to modulate several of the EEG indicators measured.

### Tasks

**Fig. 1:**
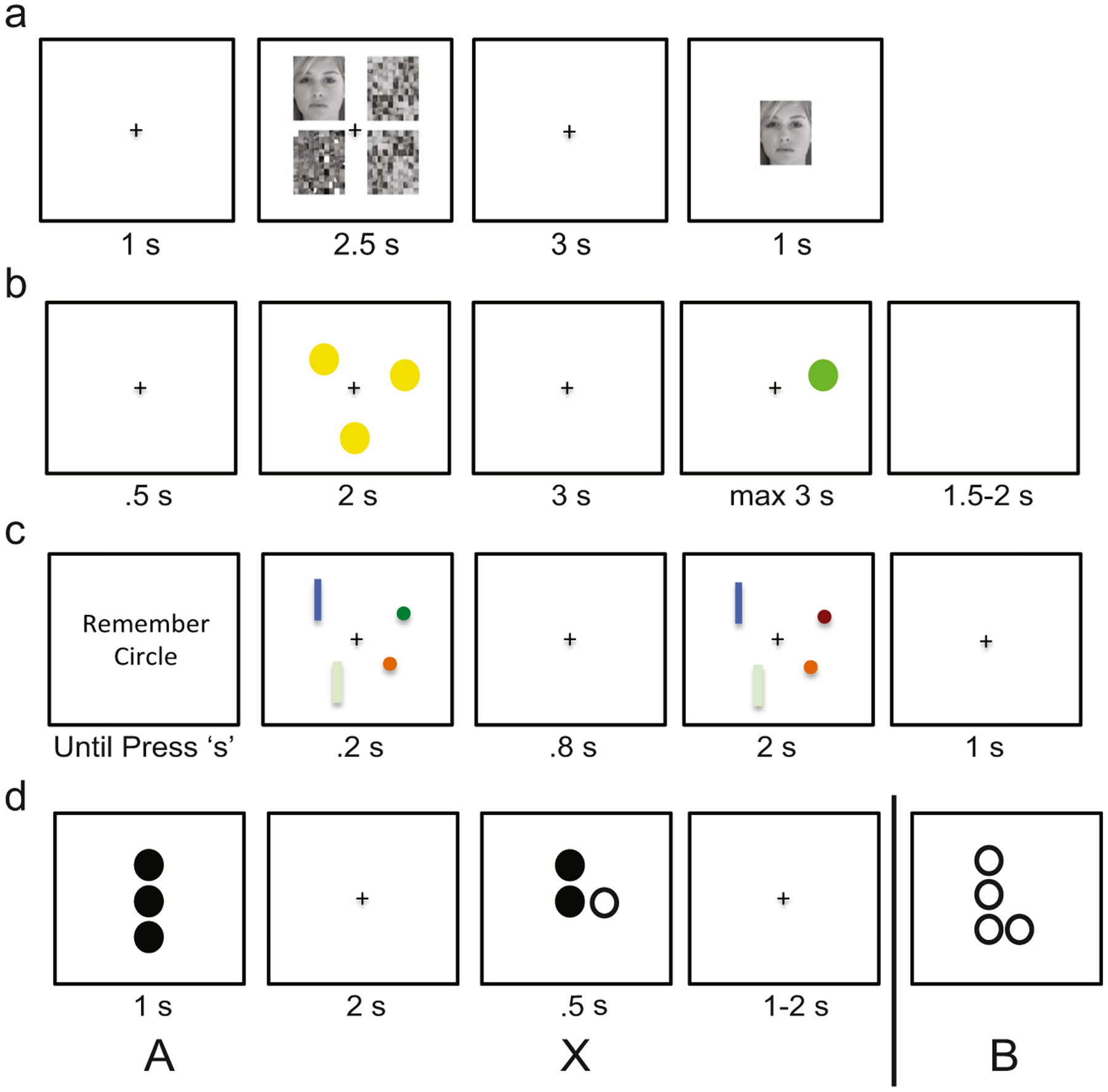
Study participants performed four working memory tasks during which EEG signals were acquired: **(a)** delayed face recognition (DFR), **(b)** Sternberg spatial working memory (SWM), **(c)** lateralized change detection (LCD), and **(d)** dot-pattern expectancy (DPX) task. Each task included a target-stimulus encoding phase, a maintenance phase during which the target disappeared off screen, and a probe phase during a response was recorded. During the DPX task target and probe were defined by stimulus pairing. Namely, participants were to respond if a stimulus labeled as X, followed a target labeled as “A”, but not following any other stimulus (i.e., “B”). See text for additional details

Participants completed four different WM tasks during which EEG was recorded: lateralized change detection (LCD), Sternberg spatial working memory (SWM), delayed face recognition (DFR), and dot-pattern expectancy task (DPX) (Figure 1). The tasks were presented in one of two orders, counterbalanced across participants (SWM, LCD, DPX, DFR or DFR, DPX, LCD, SWM). For all tasks, stimuli were presented on a Dell PC (Round Rock, TX; 17” monitors with 4:3 aspect ratio) and responses were collected on a QWERTY keyboard, controlled by E-Prime Software (v2.0; Psychology Software Tools, Pittsburg, PA).

We note that tasks involving overt verbal responses were not included, because tasks with these response characteristics are generally less suitable for and therefore not as well validated as the selected tasks that demand primarily button-press responses and have been validated in functional MRI paradigms. We additionally aimed to minimize the use of verbal task stimuli to avoid possible confounding of specific WM processes with individual differences in semantic knowledge between participants (given that those with more robust semantic capacities would require less WM to maintain the same material in mind). A larger battery of tasks, including other complex WM tasks and verbal WM tasks was completed separately from the EEG sessions. To ensure the tasks were consistent with current definitions of working memory constructs, the tasks were selected from published recommendations of two current initiatives: CNTRICS - *Cognitive Neuroscience Treatment Research to Improve Cognition in Schizophrenia* (Barch, Berman, et al., 2009a; Barch, Carter, et al., 2009b); and RDoC – *Research Domain Criteria* (Bilder et al., 2019; Insel et al., 2010). Each of the EEG tasks are described below. We will first describe the tasks during which EEG was recorded and then describe further evidence that the EEG signals extracted during these tasks are related to specific circuit mechanisms, as noted above: local cortical circuits (*short-term storage)*; corticohippocampal circuits *(long-term storage)*; frontoparietal corticocortical circuits *(goal maintenance)*; and corticostriatopallidothalamic (CSTP) circuits (*goal maintenance* & *updating*).

It should be noted that there is not an isomorphic relation between individual tasks and putative circuit inferences because the circuit inference depends on when during the task the EEG signal was collected and other signal characteristics, including frequency, amplitude, and spatial location(s). For example, in the Spatial Working Memory task described below, EEG signal collected during the encoding phase is assumed to be related to different circuit mechanisms than EEG activity collecting during the maintenance period. Other specific signals (e.g., event-related decreases in alpha power, or the P3b event-related potential) also merit unique interpretations and are discussed below in the section on EEG Feature Extraction.

#### Lateralized Change Detection (LCD)

The LCD task uniquely captures the contralateral delay activity (CDA), a feature derived from the EEG signal during WM maintenance, that has been proposed as a correlate of WM capacity. The CDA also is a putative indicator of local circuit integrity, albeit there are identified moderating roles of both long range corticocortical and CSPT circuits on capacity (Leonard et al., 2013; Vogel & Machizawa, 2004). In this task, in a given trial, participants are first presented with a cue that instructs them to remember the color of either circles or rectangles, in a subsequent 200-ms visual array containing rectangles on one side of fixation and circles on the other side, with side-allocation randomly varied across trial. The array is followed by an 800-ms maintenance period during which only a fixation marker is present on screen. Finally, a target array is presented that is either identical to the encoding array or differs via a color change of one of the attended elements (unattended elements were unchanged). Participants have 2,000 ms to indicate by button press whether the target is same or different than the encoding array. For each participant, trials varied randomly in attended shape (circles/rectangles) and whether the target matched/did not match the encoding array, with equal proportions of each trial type per subject. Furthermore, a given trial varied in load, namely the number of elements to remember (1, 3, or 5). A total of 36 trials were presented per block and participants performed a total of 6 blocks (216 total), resulting in 72 trials per load. Our primary behavioral outcome was maximum storage capacity (Leonard et al., 2013) (K_LCD_), defined as K = *n* * (HR-FA)/(1-FA), where *n* is the number of to-be-remembered objects, HR is the hit rate, and FA is the false alarm rate; the maximum value across all values of n was used. We also recorded median reaction time (RT_LCD_) and standard deviation of RT (SDRT_LCD_) for correct trials.

#### Spatial Working Memory (SWM)

The SWM task is a delayed match-to-sample variant of the classic “Sternberg” paradigm that is an archetypal measure of WM “maintenance” thought to depend on frontoparietal corticocortical and CSPT circuits (Glahn et al., 2002). Trials begin with a fixation cross presented for 500-msec, followed by a 2-sec encoding display containing 1, 3, 5, or 7 yellow dots whose locations are to be remembered. The number of dots is a manipulation of load, with greater load expected to engage more WM. The screen then turns blank for a 3-sec maintenance interval. A single probe dot then appears and remains onscreen for a maximum of 3 sec. During this time, participants indicate with a button press (left and right arrows) whether this probe stimulus is in a location previously shown (match) or not (non-match). A blank screen follows for a 1.5- to 2-sec intertrial interval. For each participant, trials vary randomly in load and whether a match or non-match probe is presented, with equal number of trials per condition per subject. Participants perform a total of 96 trials (24 per load), presented in 4 blocks of 24 trials (with equal number of trials per condition per block). Primary behavior outcome variables are maximum capacity, as well as reaction time (RT_SWM_) and its standard deviation (SDRT_SWM_) for correct trials. Capacity was defined following Cowan as, K = *n* * (H-FA), where *n* = load, H = hit rate, FA = false-alarm rate. Maximum capacity (K_SWM_) was defined as the maximum capacity across loads.

#### Delayed Face Recognition (DFR)

The DFR involves presentation of novel face stimuli in a SWM task delayed match-to-sample format; this task has been used to identify corticohippocampal circuit activations recruited during conditions of high memory load (Rissman, Gazzaley, & D’Esposito, 2008). Each trial begins with a 2.5-sec encoding stimulus that consists of four images presented simultaneously, one in each quadrant around the fixation cross. The four images include either 1 or 3 face stimuli, reflecting low or high load, interspersed with scrambled faces. Following a 3-sec delay during which a fixation cross is presented, a probe face appears for 1 sec, and subjects respond indicating whether or not it matches any of the initial faces (using left and right arrow keys). A screen containing only a fixation cross then appears for a 1-sec interstimulus interval. Trials vary randomly in load, gender of face stimuli, and whether a match or nonmatch probe is presented, with equal number of trials per condition. The trial order was generated once, and the same sequence was used for all participants. Participants perform a total of 96 trials (48 per load), presented in 3 blocks of 32 trials. Primary behavior outcome variables are maximum capacity during load 3 (K_DFR_), as well as reaction time (RT_DFR_) and its standard deviation (SDRT_DFR_) for correct trials. Capacity is defined as for the SWM task.

#### Dot Probe Expectancy (DPX)

The DPX is a continuous performance task, developed as a variant of the widely validated AX-CPT task (Barch et al., 2004; Cohen, Barch, Carter, & Servan-Schreiber, 1999), and was selected to assess WM goal maintenance. The task has undergone fMRI validation, demonstrating replicable frontoparietal corticocortical activations (Barch, Moore, Nee, Manoach, & Luck, 2012; Poppe et al., 2016). The DPX variant was developed as a clinical tool (Henderson et al., 2012), with the original letter stimuli of the AX-CPT task replaced by spatial dot stimuli, and the number of trials and interstimulus interval duration optimized to minimize testing time while maintaining sensitivity. Trials in this task comprise pairs of stimuli, context and target, that can be one of four types denoted AX, AY, BX, and BY. Participants are to respond (button press) during the target only for AX trials, namely if the context is stimulus A and the target is stimulus X. Any response for trial types AY, BX, or BY is an error. The task thus assesses the extent to which the context (i.e., A or B stimulus) is effectively maintained to guide response to the target (i.e., X or Y stimulus). A trial begins with presentation of the context stimulus for 1 sec, followed by a 2-sec maintenance interval during which only a fixation cross is on screen. The target then appears for 500 ms. The interstimulus interval follows, with duration drawn randomly from a square distribution (min = 1 sec, max = 2 sec). Participants perform a total of 216 trials, presented in 6 blocks. In each block, there are 26 AX trials, 4 AY and BX trials and 2 BY trials, presented in a randomized order, for a total of 156 AX trials, 24 AY/BX trials, and 12 BY trials. The trial order was generated once, and the same sequence was used for all participants. Behavioral outcome measures included *d’*_DPX_ (d-prime), as well as reaction time (RT_DPX_) and its standard deviation (SDRT_DPX_) for correct AX trials. D-prime was defined as z(HR)-z(FA), where z(HR) is the z transform of AX hit rate and z(FA) is the z transform of AX false alarm rate. This task thus was an outlier in its measure of WM integrity. Rather than measuring capacity via load manipulation, WM integrity was quantified through signal detection sensitivity (detection of X), which is dependent on maintenance of context (A).

### EEG Recording

While participants performed each of the four WM tasks, EEG recordings were collected using BioSemi Active Two system, containing 64 silver chloride electrodes positioned in accordance with the 10/20 System. No filters were applied during acquisition – the Active Two system acquires fully DC coupled signals. Electrode impedances were brought below 20KΩ before task recording. Electrical signals were recorded using BioSemi hardware and ActiView recording software (BioSemi B.V., Netherlands). EEG was recorded at 1024 Hz with no reference (as implemented in BioSemi’s zero-ref framework). Electrode locations were recorded prior to the EEG session using a Zebris digitizer (Zebris Medical GmbH, Germany).

### Behavioral Dimension Analysis

The analysis included the 12 behavioral outcome measures for the four tasks: median RT and its standard deviation (DPX, DFR, SWM, DPX), as well as WM capacity (DPX, DFR, SWM) and d’ (DPX). The reaction time (RT) measures are not always included in studies of WM but have been related to goal updating and interference control (Kessler & Oberauer, 2015); specifically, RT tends to be slower when updating demands increase and faster when participants master task demands and effective costs of updating lessen. The measures of WM capacity are widely used directly to indicate the capacity of the relevant neural systems to represent and maintain the task-relevant information (Barch et al., 2012). D-prime has been used widely to index freedom from interference (Haatveit et al., 2010). Because our goal was to obtain performance metrics representative of WM processes independent of task, our first objective was to identify common underlying dimensions across these 12 measures. To do so, we performed a principal components analysis. Model assumptions were tested using Kaiser-Meyer-Olkin Measure of Sampling Adequacy (>0.6) and Bartlett’s Test of Sphericity (*p* < 0.0001). The number of factors was selected automatically based on eigenvalue >1. Factors were rotated using Promax oblique rotation, selected based on the presence of factor correlations >|0.32|, (*r* = −0.41) (Tabachnick & Fidell, 2007). Factor scores derived from this analysis served as outcome variables in the regression models below (e.g., *EEG Predictors of Behavioral WM Outcomes*).

### EEG Preprocessing

The EEG signals collected during the WM tasks were processed using custom MATLAB (The Mathworks, Inc.) scripts calling functions from the EEGLAB (v.11.03.b) software (Delorme & Makeig, 2004). Continuous EEG data were high pass filtered (>1 Hz), inspected for noisy electrodes, which were excluded from further analysis. It is notable that the 1-Hz high pass was selected, because it improves the quality of the data and component decomposition (Winkler, Debener, Muller, & Tangermann, 2015). We acknowledge that high-pass filters >0.3 Hz can distort the ERP (Tanner, Morgan-Short, & Luck, 2015; Tanner, Norton, Morgan-Short, & Luck, 2016); however, as we were evaluating effects with strong priors on topography, latency and condition effects, we felt the benefit to the signal-to-noise justified any potential distortions. The data were re-referenced to average reference. Within each subject, epochs of gross movements and muscle artifact were identified and removed if signal power in that epoch exceed the 85^th^ percentile for >60% of the channels. The cleaned data were decomposed into source signals by independent component analysis (ICA) using the *binica* program in EEGLAB (extended *infomax* algorithm, stopping weight change set to 1e-7, maximum learning steps set to 1000, see S2 in Supplemental Materials for additional details) (Lee, Girolami, & Sejnowski, 1999). Each IC time course is thought to reflect a putative cortical source generator, associated with a single topography across electrodes. IC time courses were analyzed in lieu of channel data in all subsequent analyses (unless specifically noted), segmented into epochs time-locked to the onset of the encoding stimulus, as described in the subsequent section. Except where stated, we restricted analysis to IC time courses with frontal and occipital topography. We excluded other ICs for two reasons. First, frontal and occipital topography ICs encompass the range of topographies of the EEG features analyzed. Related to this point, frontal and occipital ICs were also identified in most subjects’ data (n = 153). In contrast other IC types were inconsistently present across subjects, which would reduce the sample size of the final analysis. Features of interest were identified and extracted from IC time courses of each subject based on a priori criteria as follows.

### EEG Feature Extraction

**Fig. 2:**
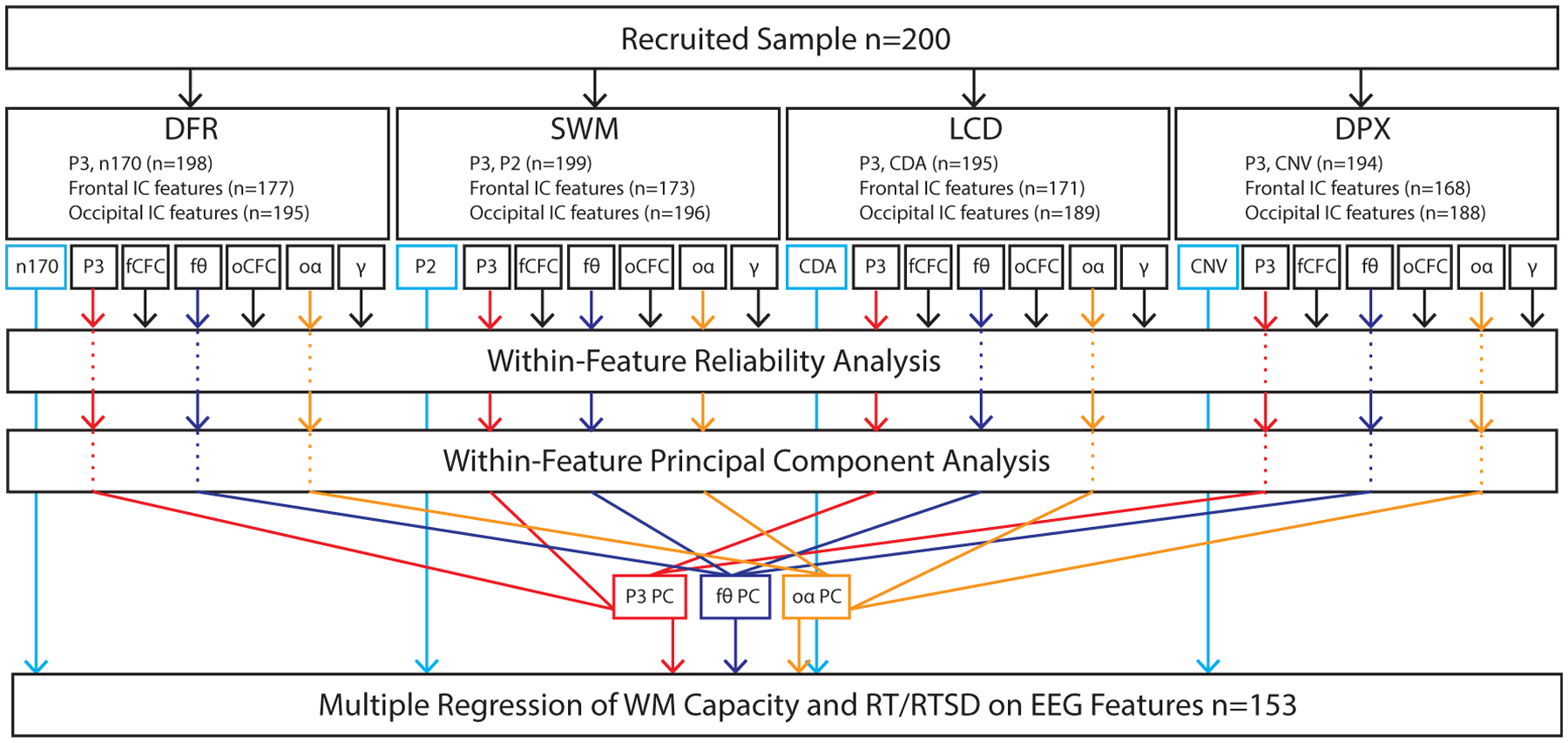
Data analysis overview. Each participant performed four EEG tasks, from which we extracted several features—associated with putative WM subprocesses (Table 4). Frontal-topography independent component (IC) features were not available in all datasets, contributing to sample size reduction. Features that were sampled across multiple tasks (P3b, fCFC, fθ, oCFC, oα, γ) were tested for reliability and then reduced via principal component analysis to a single dimension or excluded from subsequent steps if threshold was not met (*Cronbach’s alpha* ≤ 0.5). Features that were unique to a given task (N170, P2, CDA, CNV) were automatically included in the final analysis. The final multiple regression was performed for datasets that contained all features (n = 153). A separate analysis was performed for WM capacity and RT/RTSD. fCFC = frontal cross-frequency coupling; fθ = frontal theta; oCFC = occipital cross-frequency coupling; oα = occipital alpha; γ = gamma; CDA = contralateral delay activity; CNV = contingent negative variation

The EEG data were used to extract features hypothesized, based on current literature, to measure elements of three core functional subprocesses contributing to WM maintenance: local cortical storage in short-term memory, long-term storage processes presumed to rely on cortico-hippocampal interactions, and goal maintenance/interference control thought to involve fronto-parietal circuitry. We also quantified a key putative supporting sub-process, namely WM updating, that complements the WM maintenance process. Additionally, we explored the contribution of vigilance-related alerting responses to WM performance. We note that all features were identified at the outset of the analysis. As noted below, dimension reduction steps were performed with automatic selection criteria, to eliminate experimenter bias. A summary of all of the measures, and subsequent steps of dimension reduction and final selection, is presented in Figure 2 (also Table 4 in Results).

#### Short-Term Storage

To quantify local cortical processing thought to underly storage in short-term memory, we computed power in the gamma band (30–50 Hz), from ICs with frontal and occipital topography, during the maintenance interval of each task. Gamma power cortical activity has been reliably observed during WM maintenance (Roberts, Hsieh, & Ranganath, 2013), used to decode WM content (Polania, Paulus, & Nitsche, 2012), and associated with WM capacity (Sauseng et al., 2009), putatively linking this feature with a local storage function (Lisman & Buzsaki, 2008; Lisman & Idiart, 1995). For each frequency and time point in an interval, the power was divided by the mean power during baseline (−900 to −300 ms) and log-transformed (10log10) to decibel (dB) units. These values were averaged across all frequencies and time points to produce a single value per subject per task (4 for frontal topography ICs and 4 for occipital topography ICs). In addition, for the LCD task data, we computed the contralateral delay activity (CDA), a feature derived from the EEG signal during WM maintenance, that has been well validated as a correlate of WM capacity (Leonard et al., 2013; Vogel & Machizawa, 2004). Finally, we explored the amplitude of the N170 event-related potential (ERP) component during DFR encoding, averaged across electrodes O1 and O2. We hypothesized that, because of the documented relationship of N170 to face processing (Rossion & Corentin, 2011), this feature may be directly related to local storage of face encoding stimuli in this task. Amplitudes in a 60 ms window around the N170 peak were averaged across epochs, and the mean voltage in the baseline preceding the fixation cue (−200 ms to −150 ms) was subtracted. In sum, we obtained a total of 10 putative indices of short-term WM storage.

#### Long-Term Storage

To quantify long-term storage, associated with cortico-hippocampal interactions, we computed power in the theta band (3–7Hz) from ICs with frontal topography, during the maintenance interval of each task. Theta power increases during WM maintenance in proportion to WM load (Jensen & Tesche, 2002; Mitchell, McNaughton, Flanagan, & Kirk, 2008; Onton, Delorme, & Makeig, 2005) and has been proposed to play a significant role in memory encoding, in particular with respect to cortico-hippocampal interactions (Lisman & Buzsaki, 2008; Lisman & Idiart, 1995). Furthermore, the strength of cross-frequency-coupling (CFC) between theta phase and gamma amplitude is a predictive neurophysiological marker of WM (Axmacher et al., 2010; Canolty et al., 2006). As above, we calculated frontal gamma power for each frequency and time point in the maintenance interval, the power was divided by the mean power during baseline (−900 to −300 ms) and log-transformed (10log10) to decibel (dB) units. These values were averaged across all frequencies and time points in the time-frequency window to produce a single value per subject per task. To calculate theta-gamma CFC, we adapted the methods of Daume et al. (2017) to quantify phase-amplitude coupling using the Kullback-Leibler distance-based modulation index (MI) introduced by Tort et al. (2010). In addition, to impose a normalization factor on CFC values, the MI values were converted to z-scores using a surrogate distribution following the method of Canolty et al. (2006). Theta-gamma CFC MIs were calculated at both frontal and posterior ICs. In sum, we obtained a total of 12 putative indices of long-term WM storage (frontal theta for each of 4 tasks, theta-gamma CFC for frontal and occipital ICs for each of 4 tasks).

#### Goal Maintenance

To quantify goal maintenance and control, thought to engage fronto-parietal circuitry, we measured CFC between alpha range (8–12 Hz) phase and gamma amplitude during the maintenance phase of each task, across ICs with frontal or occipital topography. The motivation for this metric stems from animal and human research supporting an association between alpha range signals and engagement of the attention system (Foxe & Snyder, 2011; Jensen & Mazaheri, 2010; Klimesch, 1997), as well as the analogous theoretical framework suggesting that such alpha-range attentional signals modulate gamma-range neuronal firing (Griffiths et al., 2019; Leszczynski, Fell, & Axmacher, 2015; Roux & Uhlhaas, 2014). Alpha-gamma CFC MI’s were calculated as described for theta-gamma CFC (*c.f. Long-Term Storage*), replacing theta phase, with alpha phase. In addition, for the DPX task, we calculated the mean amplitude, at electrode FCz, of the contingent negative variation (CNV) ERP, in the 100 ms immediately preceding target onset. The CNV is a slow, negative potential that occurs when expecting a target that is contingent on a prior stimulus (i.e., as X is contingent on A in DPX). It is thought to include motor and cognitive operations necessary to prepare for the expected event, with numerous cortical sources in frontal cortex, associated with core goal maintenance functions (Fuster, 1985; Rosahl & Knight, 1995). In sum, we extracted 9 features (alpha-gamma CFC across 4 tasks, in frontal and occipital ICs, as well as CNV at FCz).

#### WM Updating

In addition to core WM maintenance subprocesses, we sought to evaluate supporting functions that are arguably critical to effective WM performance that are either conceptualized as different from or complementary to maintenance operations. This includes updating and alerting; we have written elsewhere about the likelihood that these functions use common phasic “arousal” mechanisms to introduce flexibility into stimulus processing and response selection (Bilder, 2012). Updating refers to subprocesses that occur during stimulus encoding, when information entering via the visual system must interact with the WM system to introduce new material for storage or to select a stimulus- and task-relevant response. We identified two EEG features to quantify this function, alpha signal power decreases and P3b ERP amplitude during encoding and probe stimuli. As noted above, alpha-range oscillatory signals are strongly implicated in the gating function of the attention system, that controls which stimuli are processed and which stimuli are ignored or gated out from further processing (Foxe & Snyder, 2011; Jensen & Mazaheri, 2010; Klimesch, 1997). During stimulus processing, event-related decreases (ERDs) in alpha power strengthen with degree of processing (e.g., encoding with load, visual detection) and accuracy of subsequent behavioral response (e.g., recall accuracy, detection accuracy) (Hanslmayr et al., 2005; Romei et al., 2008; Samaha & Postle, 2015), suggesting that alpha ERDs play an important role in WM updating (Klimesch, 1997, 2012). Alpha ERD during encoding and probe stimuli in each task were therefore calculated, following protocols as described for gamma (*c.f., Short-Term Storage*) and theta (*c.f., Long-Term Storage*), for ICs with occipital topography. Note that because we found alpha ERD was significantly correlated across encoding and probe events in all tasks (*r*_*DPX*_ = 0.80, *r*_*SCAP*_ = 0.72, *r*_*DFR*_ = *0.56, r*_*LCD*_ = 0.80, *p* < 0.0001), we additionally collapsed alpha ERD across these events. Finally, although alpha is typically defined as 8–12Hz, we have previously found that the full span of the effect extends upwards through approximately 16 Hz (Lenartowicz et al., 2014; Lenartowicz et al., 2019). Hence, we defined the alpha band as 12–16 Hz. This was validated by confirming that 8–12 Hz and 12–16 Hz ERD values are significantly correlated during both encoding (*r*_*DPX*_ = 0.74, *r*_*SCAP*_ = 0.70, *r*_*DFR*_ = 0.66, *r*_*LCD*_ = 0.60, *p* < 0.0001) and probe (*r*_*DPX*_ = 0.70, *r*_*SCAP*_ = 0.73, *r*_*DFR*_ = 0.68, *r*_*LCD*_ = 0.65, *p* < 0.0001) events.

The second feature related to WM updating that we identified was amplitude of the P3b ERP. Maximal over parietal scalp around 300–600 ms following stimulus onset, this positive potential has been associated with the function of updating context in WM (Polich, 2007) and, in alternate formulations, with categorization of content within WM (Kok, 2001; Nieuwenhuis, Aston-Jones, & Cohen, 2005). The P3b thus is directly related to encoding processes that interact with WM. To quantify the P3b, we identified the P3b peak in the 300–600 ms window during each encoding and probe stimulus, and calculated the mean amplitude in the 50-ms window around the peak at electrode Pz, selected based on known priors (Lenartowicz, Escobedo-Quiroz, & Cohen, 2010). As in the case of alpha ERD, P3b amplitude was significantly correlated across encoding and probe events in all tasks (*r*_*DPX*_ = 0.81, *r*_*SCAP*_ = 0.55, *r*_*DFR*_ = 0.78, *r*_*LCD*_ = 0.74, *p* < 0.0001), hence we collapsed P3b amplitude values across these events.

Finally, we quantified alerting, as an exploratory index of general vigilance processes, reasoning that if participants are disengaged from the task, their WM maintenance and consequently performance also may be compromised. We quantified alerting via P2 ERP amplitude, in response to a fixation stimulus, present at the onset of each trial in the SWM task. In prior studies, the P2 has been shown to increase for cues of increasing complexity (Lenartowicz et al., 2010), stimuli of increasing task relevance (Potts, 2004) and during fixation for individuals with higher WM performance (Lenartowicz et al., 2014), suggesting that it may be a relevant indicator of an alerting response at the onset of behaviorally important events. P2 amplitude was calculated at electrode Fz as the average voltage in the 50-ms window surrounding the peak in the 150–250 window post-fixation. Thus, we obtained 9 additional EEG measures, 8 of WM updating (alpha ERD in each of 4 tasks, P3b in each of 4 tasks), and also P2 during fixation in the SWM task.

### EEG Dimension Reduction

In sum, we extracted a total of 40 EEG features, some of which were repeated across tasks (e.g., gamma or theta during maintenance) and others that were unique (e.g., CDA in LCD task or CNV in DPX task). In order to reduce the number of variables, we conducted additional principal component analyses for each EEG feature that was present in all four tasks (Figure 2). This included gamma (c.f., *Short-Term Storage*), theta and theta-gamma CFC (c.f., *Long-Term Storage*), alpha-gamma CFC (c.f., *Goal Maintenance & Control*), as well as alpha ERD and P3b (c.f., *WM Updating & Alerting*). As for behavioral dimension reduction, model assumptions were tested using Kaiser-Meyer-Olkin (KMO) Measure of Sampling Adequacy (>0.6) and Bartlett’s Test of Sphericity (*p* < 0.0001). The number of factors was selected automatically based on eigenvalue >1, and only one PCA was run per cluster of measures, eliminating experimenter bias at this stage. Factors were rotated using Promax oblique rotation, to allow for the presence of factor intercorrelations. This analysis was critical in testing whether the proposed subprocesses of WM are common across multiple tasks, in which case we would expect a single principal component for a given metric to load on all four tasks. Finally, to further test the validity of the WM subprocesses, we also performed a reliability analysis across the four tasks, for each of the above metrics, eliminating from further analysis any indicators with *Cronbach’s alpha* < 0.5. This is a liberal threshold, without prior expectation on what a sufficient reliability threshold might be for these metrics and chosen with the goal of eliminating highly unreliable features. The final set of predictors produced by this protocol is summarized in Table 4 and Figure 2.

### EEG Predictors of Behavioral WM Outcomes

The core question driving this study was to determine which sub-processes of WM, as quantified by select EEG features, predict performance in WM. To address this question, we performed a confirmatory multiple regression analysis (SPSS v.25, IBM Corporation, Somers, NY). A separate analysis was conducted for each outcome variable, namely, component scores derived from the behavioral dimension PCA. As predictors, we included all EEG measures extracted for each of the putative WM sub-processes described above. Covariate regressors included age, gender and sampling group (care seeking versus non-care seeking), included to account for confounding effects. Individual coefficients were evaluated using *t*-tests. The final sample size in this analysis is 153. As shown in Figure 2, data loss resulted from (a) initial loss due to issues with EEG data collection, and (b) exclusion of individuals who did not show *both* frontal and occipital ICs in the decomposition, meaning that all predictors could not be represented in the model. These were treated as missing data and excluded in the multiple regression. Finally, regression coefficients at *p* < 0.05 are interpreted as significant. We do not apply multiple comparison correction (to account for two models, one for each performance dimension) as they address, in principle, independent behavioral dimensions. Further, no corrections are warranted at the dimension reduction steps, as no inference was performed.

### Load and Group Effects

To further support interpretation, we performed a repeated-measures ANOVA for each measure of interest within each task, with independent variables including the within-subject variable LOAD (low, high, varying by task), as well as the between-subject variable sampling GROUP (care-seeking vs. non-care seeking). A significant LOAD main effect was important to validate the role of a given EEG feature in WM capacity, because capacity is defined by amount of content stored. Note that LOAD was not manipulated in the DPX task and so it was replaced by the comparison of TRIAL type (A vs. B cues) in this analysis. The GROUP variable was included as a covariate to capture differences in EEG features as a function of group differences in psychopathology. The GROUP variable validates the sampling method in this project, designed to capture a broad range of psychopathology in the CS group. Finally, we report Cohen’s *f* effect sizes, recommended for analyses involving F tests or ANOVA models (J. Cohen, 1988). Small, medium and large *f* values are traditionally defined as 0.02, 0.15, and 0.35, respectively.

## Results

### Sample Demographics

Sample demographics are shown in Table 1, task performance in Table 2. Following the sampling strategy, group membership is defined by Care-Seeking (CS) and Non-Care-Seeking (NCS) definitions rather than DSM-5 diagnoses, but the diagnoses observed in each group are shown in Table 1. The samples were comparable in age, gender distribution, years of education, estimated IQ, annual household income, and racial distribution. The sampling strategy was validated by increased overall disability scores and increased presence of positive neuropsychiatric diagnoses in the care-seeking (CS) participants relative to the non-care-seeking group (NCS). The CS group also showed lower capacity scores, slower RT and greater RT variability across several, although not all, tasks (Table 2), a pattern that was replicated in the PCA-reduced dimension scores as well (*c.f., Behavioral Dimensions* below).

#### Task Reliability

Given some variability in group differences in capacity across tasks, we sought to also evaluate the reliability of the tasks. We first calculated *Cronbach’s alpha* for each of accuracy, RT, and RTSD, across the four tasks. Reliability was good for all metrics (n = 195): Accuracy *Cronbach’s alpha* = 0.78; RT *Cronbach’s alpha* = 0.72; RTSD *Cronbach’s alpha* = 0.69. Pair-wise task correlations were significant across all pairs, and in the range of 0.35–0.62 for accuracy, 0.31–0.58 for RT, and 0.30–0.51 for RTSD.

### Behavioral Dimensions

The behavioral-measure PCA identified two components with eigenvalues >1, explaining 52% of total variance. Model assumptions were met (*KMO* = 0.73, Bartlett’s test of Sphericity, *x*^2^ = 1012, *p* < 0.0001). The rotated component loadings are shown in Table 3, with loadings >0.3 considered as making a significant contribution to the component. The first component explained 37.4% of total variance. Loadings on this component were greatest from RT-related metrics, namely RT and SDRT for all four tasks. A higher score on this component would indicate slower and more variable responding. The second component explained 14.5% total variance. Strongest loadings on this component corresponded to capacity indices (K) across tasks. Higher scores on this component would indicate higher capacity across tasks. Thus, the analysis identified capacity and RT/RTSD as separable, albeit correlated (*r* = −0.41), dimensions across four WM tasks. Individual scores on these components were extracted via regression and used as WM outcomes below, with component 1 scores referred to as a composite RT/SDRT outcome variable and component 2 scores referred to as a composite capacity (K) outcome variable. A validation *t*-test analysis indicated that composite RT/SDRT was faster/lower (*t*(193) = 3.1, *p* = 0.002, *M* = −0.33 vs. 0.14), and composite capacity was higher (*t*(193) = 2.5, *p* = 0.01, *M* = 0.27 vs. −0.11), in NCS individuals than CS individuals.

### Working Memory EEG Features

**Fig. 3:**
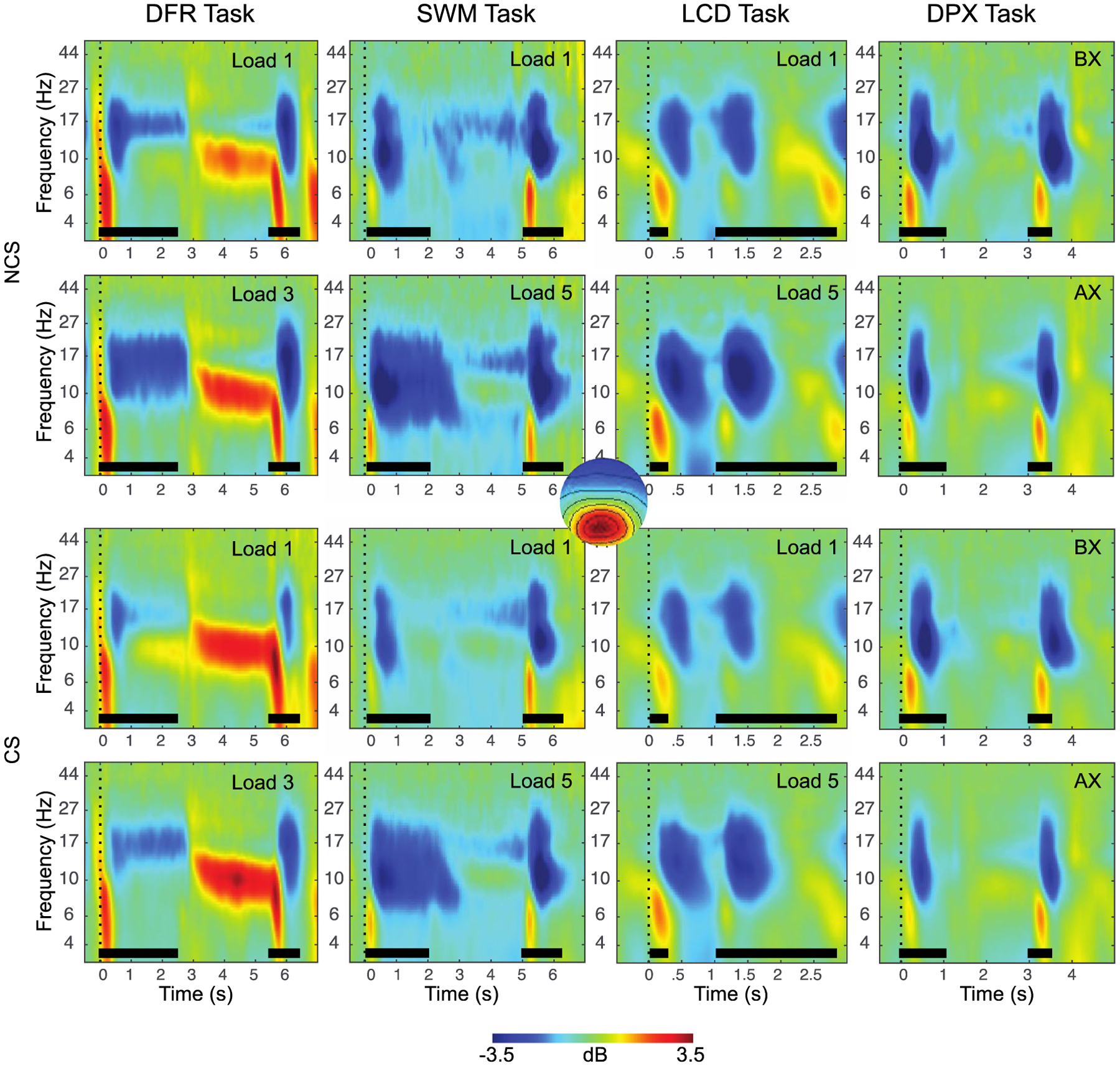
Event-related changes in spectral power are shown for each task, across the trial window, expressed in dB scale relative to the prestimulus baseline. The plots show responses for independent components with occipital topography. Inset at center shows this topography, with red indicating electrodes maximally contributing to the IC timeseries. The duration of the encoding target and probe stimulus is indicated with a black bar in each plot. The top two rows show exemplary low-load and high-load conditions for non-care-seeking (NCS) individuals, the bottom two rows show exemplary low-load and high-load conditions for care-seeking (CS) individuals. Clear decreases in power are apparent during each stimulus across 8–20 Hz (blue), with the DFR task also showing increases in power during the maintenance period. These EEG features also show clear load (greater at high load) and group (more negative in NCS) effects. DFR = delayed face recognition, SWM = Sternberg spatial working memory; LCD = lateralized change detection; DPX = dot-pattern expectancy

**Fig. 4:**
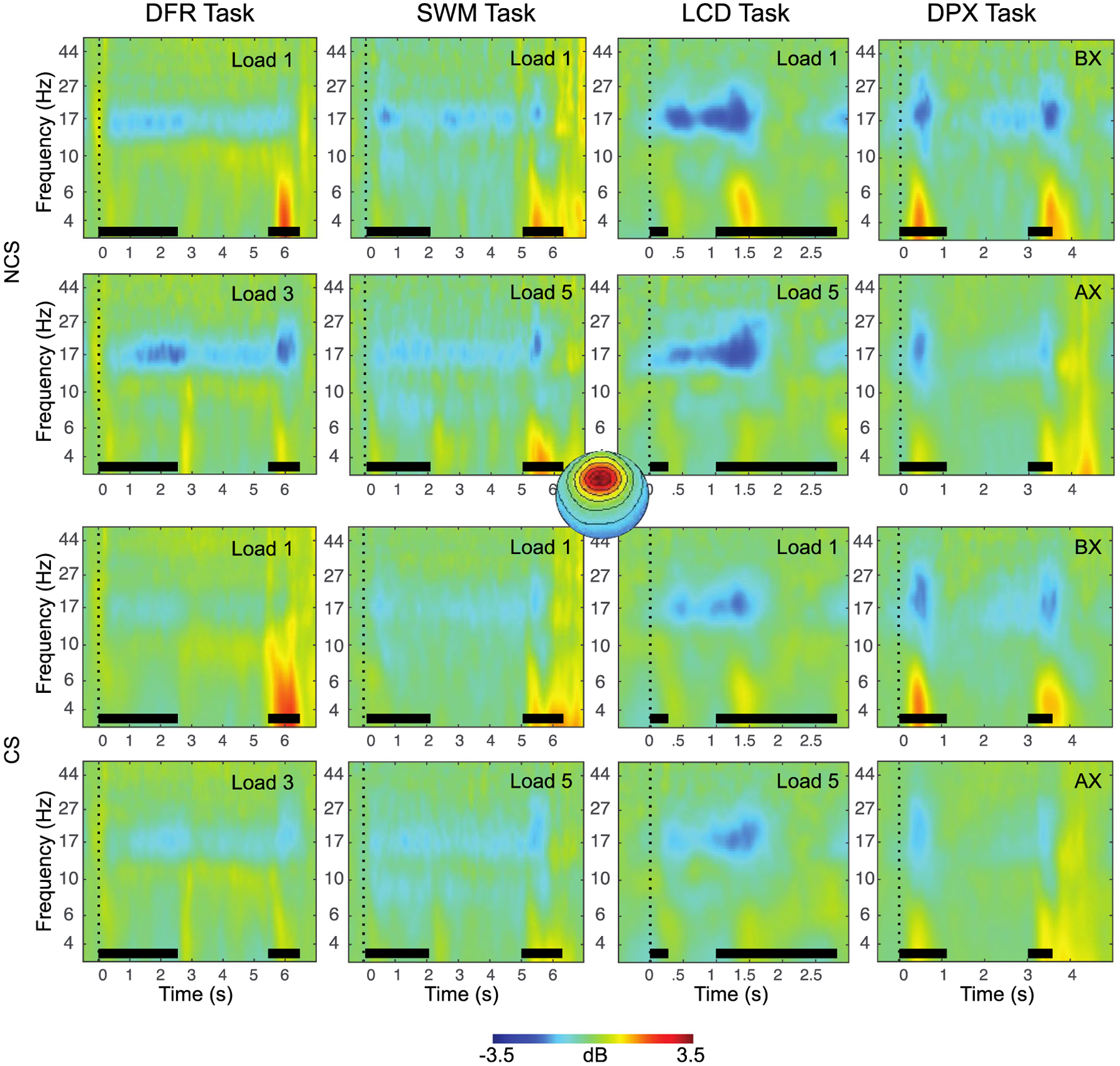
Event-related changes in spectral power are shown for each task, across the trial window, expressed in dB scale relative to the prestimulus baseline. The plots show responses for independent components with frontal topography. Inset at center shows this topography, with red indicating electrodes maximally contributing to the IC timeseries. The duration of the encoding target and probe stimulus is indicated with a black bar in each plot. The top two rows show exemplary low-load and high-load conditions for non-care-seeking (NCS) individuals, the bottom two rows show exemplary low-load and high-load conditions for care-seeking (CS) individuals. Stimulus events (black bars) are synchronous with power increases (4–8 Hz) and decreases (15–20 Hz) that vary in degree across tasks and sampling groups. DFR = delayed face recognition; SWM = Sternberg spatial working memory; LCD = lateralized change detection; DPX = dot-pattern expectancy.

**Fig. 5:**
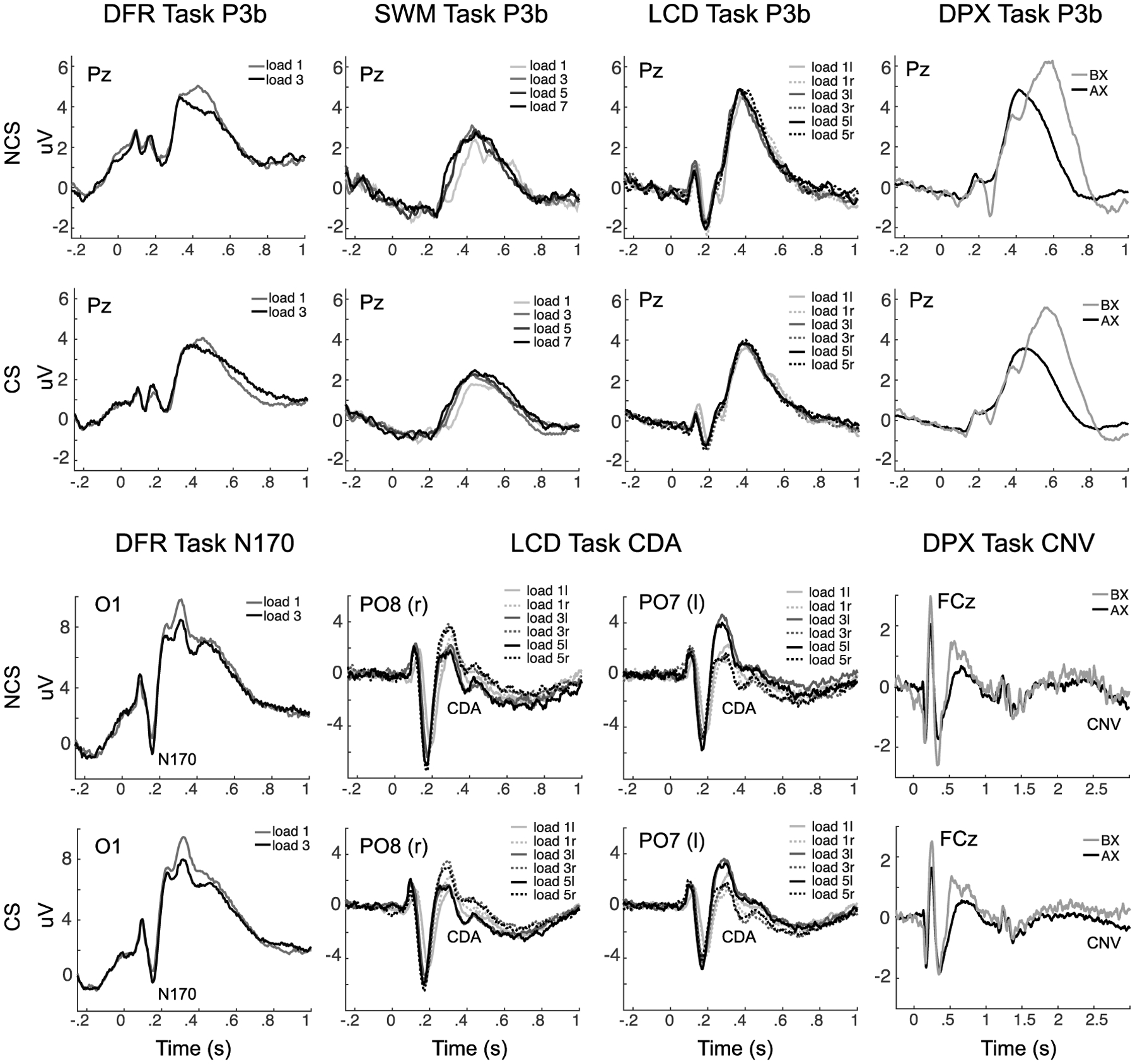
Event-related changes in potential are shown for each task, across the trial window, expressed relative to the prestimulus baseline. Plots shown for select electrodes. The top two rows show P3b responses for non-care-seeking (NCS) and care-seeking (CS) individuals across all four tasks. Bottom two rows show N170, CDA and CNV ERPs in DFR, LCD, and DPX respective, for NCS and CS individuals. The P3b is greater for NCS participants and shows expected decreases with load in the DFR task, but increased amplitude with load in SWM, LCD and DPX, possibly due to confounded increases in saliency. The N170 in DFR is also enhanced with higher load. The CDA is evident in the LCD task, with more negative potential (e.g., 0.2–0.6 s poststimulus) for contralateral stimuli in each hemisphere (r = right, l = left). The CNV in DPX task, at electrode FCz, is more negative in AX than BX trials consistent with expectancy of X stimuli and associated responses. DFR = delayed face recognition; SWM = Sternberg spatial working memory; LCD = lateralized change detection; DPX = dot-pattern expectancy

The final EEG features identified for each WM subprocess are summarized in Table 4 and illustrated in Figures 3, 4 and 5. A total of 7 EEG features of the original 40 were retained as predictors for subsequent analyses.

#### Short-Term Storage

We retained only CDA (LCD task) and N170 (DFR task) indicators of short-term storage. Across-task *Cronbach’s alpha* for frontal maintenance gamma was 0.50 and for occipital maintenance gamma was 0.46. Thus, gamma showed relatively poor to moderate reliability across tasks, and was not retained for further analysis (see Table S1 for PCA results for this measure).

#### Long-Term Storage

A PCA on frontal theta during maintenance across tasks identified a single component, explaining 45.5% of total variance (*KMO* = 0.49, Bartlett’s test of Sphericity, *x*^2^ = 336, *p* < 0.0001, see Table S2 for component loadings). *Cronbach’s alpha* for theta across tasks was 0.52, thus it was retained for further analysis. In contrast, *Cronbach’s alpha* for theta-gamma CFC measures was 0.06 for parietal ICs and 0.02 for frontal ICs. Thus, theta-gamma CFC measures were excluded from further analysis due to low reliability across tasks.

#### Goal Maintenance

The only EEG feature included was CNV, measured in the DPX task. This was because for alpha-gamma CFC, *Cronbach’s alpha* was 0.02 and 0.13 for frontal and posterior ICs respectively. Thus, alpha-gamma CFC measures were excluded from further analysis.

#### WM Updating

With respect to WM updating, both alpha ERD and P3b were reliable, with *Cronbach’s alpha* across tasks exceeding 0.7 (alpha ERD = 0.72, P3b = 0.72). Also, both measures resulted in a single component following PCA. For alpha ERD, a single component explained 62.5% of total variance (*KMO* = 0.75, Bartlett’s test of Sphericity, *x*^2^ = 238, *p* < 0.0001, see Table S4 for component loadings). For P3b, a single component explained 63.3% of total variance (*KMO* = 0.76, Bartlett’s test of Sphericity, *x*^2^ = 253, *p* < 0.0001, see Table S3 for component loadings). Finally, alerting was measured using P2 amplitude during fixation in SWM only; thus, no further reduction was needed.

#### EEG Predictors of WM Capacity

The results of regressing composite capacity (K) on EEG features, across WM subprocesses, is shown in Table 5. Higher composite capacity scores were associated with two EEG features, both during the maintenance interval: (1) more negative (and therefore stronger) CDA in the LCD task (*p* = 0.001), and (2) higher composite frontal theta scores (*p* = 0.008). Thus, composite capacity scores were most strongly associated with EEG indicators of short and long-term storage functions. At a more liberal false positive threshold of *p* < 0.1, higher composite capacity scores we also associated with higher P3b amplitude (*p* = 0.06), an index of WM updating, and stronger CNV in DPX task (*p* = 0.098), associated with goal maintenance.

### Load Effects

To further aid interpretation, we also evaluated effects of load on each of the significant variables, hypothesizing that features that predict WM capacity should also be sensitive to manipulations of load. These analyses were performed on non-reduced (pre-PCA) data. Of the three variables that significantly predicted composite WM capacity, we found significant load effects for CDA and inconsistent effects for maintenance theta. Namely, the main effect of load was significant for CDA (*F(2,193)* = 14.8, *p* < 0.0001, *Cohen’s f* = 0.28). As expected, magnitude of CDA increased with load (load 1: *M* = −0.16 uV, *SE* = 0.044; load 3: *M* = −0.48 uV, *SE* = 0.049; load 5: *M* = −0.40 uV, *SE* = 0.048). Frontal theta showed main effects of load in only two of four tasks, in the SWM task (*F(3,171)* = 2.6, *p* = 0.05, *Cohen’s f* = 0.13) and the LCD task (*F(2,169)* = 6.4, *p* = 0.002, *Cohen’s f* = 0.20). These effects were small in magnitude, and inconsistent in direction, with theta power lowest in low-load condition in the SWM task (*M low to high:* −0.37, −0.03, −0.19, −0.08 dB), but highest in low-load condition in the LCD task (*M low to high:* −0.14, −0.34, −0.35 dB). Thus load-effect analyses provide convergent evidence for the role of CDA, and inconsistent evidence for theta.

#### Group Effects

There was no difference in CDA between CS and NCS participants (*F(1,193)* = 0.61, *p* = 0.43, *Cohen’s f* = 0.05; CS: *M* = −0.32 uV, *SE* = 0.033; NCS: *M* = −0.37 uV, *SE* = 0.05). Similarly, groups did not differ in frontal theta: DFR (*F(1,175)* = 0.04, *p* = 0.85, *Cohen’s f* = 0.01), DPX (*F(1,166)* = 0.81, *p* = 0.37, *Cohen’s f* = .07), LCD (*F(1,169)* = 0.002, *p* = 0.96, *Cohen’s f* = 0.05), SWM (*F*(*1,171)* = 1.05, *p* = 0.31, *Cohen’s f* = 0.08).

### EEG Predictors of WM RT and SDRT

The results of regressing composite reaction time measures (RT/SDRT) on EEG features, across core WM functions, is shown in Table 5. Lower composite RT/SDRT scores (i.e., faster and less variable responding) were associated with higher P3b amplitudes (*p* = 0.006), as well as more negative (and therefore stronger) CNV in the DPX task (*p* = 0.03). Thus, composite RT/RTSD scores were most strongly associated with EEG indicators of WM updating and goal maintenance, respectively.

#### Load Effects

The P3b and CNV were also examined for load effects. The amplitude of P3b at electrode Pz during encoding showed significant load effects, although the direction varied with task. Amplitude decreased with increasing load in the DFR task, *F(1,196)* = 9.5, *p* = 0.002, *Cohen’s f* = 0.22 (*M low to high* = 4.2, 3.8 uV). In contrast, amplitude increased with increasing load in the LCD task, *F(1,193)* = 5.3, *p* = 0.005, *Cohen’s f* = 0.17 (*M low to high* = 3.8, 4.1, 4.2 uV), and the SWM task, *F(1,197)* = 4.4, *p* = 0.004, *Cohen’s f* = 0.15 (*M low to high* = 2.0, 2.6, 2.4, 2.4 uV). This reversal is likely due to known differences in task parameters (*c.f., Discussion: WM Task Differences*). In the DPX task, load was not manipulated directly; hence, instead, we evaluated effects of cue type on P3b and, also, CNV amplitude, expecting that A cues would increase demand for WM maintenance relative to B cues. The P3b amplitude, consistent with the load effects in DFR, decreased in amplitude with increasing load (*M B-cues* = 5.4 uV, *M A-cues* = 3.2 uV), *F(1,192)* = 115.4, *p* = 0.0001, *Cohen’s f* = 0.78. Amplitude of CNV was as expected, larger for A-cues than B-Cues (*M B-cues* = 0.08 uV, *M A-cues* = −0.58 uV), *F(1,192)* = 21.6, *p* = 0.0001, *Cohen’s f* = 0.34. In sum, load-effect analyses provide convergent evidence for the role of P3b, and CNV in WM RT/SDRT.

#### Group Effects

Significant effects were observed in both CNV and P3b across all tasks, with the CS group showing consistently weaker CNV/P3b than the NCS group: CNV *(F(1,192)* = 6.7, *p* = 0.01, *Cohen’s f* = 0.19) (*CS: M* = −0.11 uV, *SE* = 0.06; *NCS: M* = −0.39 uV, *SE* = 0.09); P3b in DPX *(F(1,192)* = 3.6, *p* = 0.06, *Cohen’s f* = 0.14) (*CS: M* = 4.0 uV, *SE* = 0.17; *NCS: M* = 4.6 uV, *SE* = 0.26); P3b in DFR *(F(1,196)* = 3.2, *p* = 0.08, *Cohen’s f* = 0.13) (*CS: M* = 3.6 uV, *SE* = 0.23; *NCS: M* = 4.4 uV, *SE* = .35); P3b in LCD *(F(1,193)* = 8.0, *p* = .005, *Cohen’s f* = 0.20) (*CS: M* = 3.5 uV, *SE* = 0.19; *NCS: M* = 4.5 uV, *SE* = 0.30); and P3b in SWM *(F(1,197)* = 3.4, *p* = 0.065, *Cohen’s f* = .14) (*CS: M* = 2.1 uV, *SE* = 0.14; *NCS: M* = 2.6 uV, *SE* = 0.21).

### Clinical Covariates

Next, we examined the robustness of the significant EEG predictors of WM capacity and RT/RTSD to clinical covariates. We re-ran the multiple regression analyses including one of: WHODAS overall disability index (Model A), depression and anxiety subscale theta scores for the BRIEF scales (Model B), or depression and anxiety scores for the PROMIS scales (Model C). The significant predictors in both RT/RTSD and capacity analyses were unchanged across all three models in all but one case.

#### Predictors of WM Capacity

The EEG regression coefficient for CDA remained significant across all three models (Model A: *t*(153) = −2.5, *p* = 0.005; Model B: *t*(153) = −2.4, *p* = 0.02; Model C: *t*(153) = −2.0, *p* = 0.05). The EEG regression coefficient for frontal theta remained significant across Models A and B models (Model A: *t*(153) = 2.5, *p* = 0.01; Model B: *t*(153) = 2.6, *p* = 0.01) but not Model C (*t*(153) = 1.5, *p* = 0.1).

#### Predictors of RT/RTSD

The EEG regression coefficient for CNV remained significant across all three models (Model A: *t*(153) = 2.0, *p* = 0.05; Model B: *t*(153) = 2.1, *p* = 0.04; Model C: *t*(153) = 2.1, *p* = 0.03). The EEG regression coefficient for P3b also remained significant across all three models (Model A: *t*(153) = −2.4, *p* = 0.02; Model B: *t*(153) = −2.9, *p* = 0.005; Model C: *t*(153) = −2.5, *p* = 0.02).

Additionally, the regression coefficients for covariate predictors were not significant, suggesting the observed relationships between EEG features and WM capacity or RT/RTSD are not accounted for by shared variance between EEG features and clinical covariates. The first-order correlations between EEG features and clinical covariates were largely not significant (Table S5), with the exception of P3b and less reliably for CDA and CNV, all of which weakened in magnitude as indicators of anxiety and depression increased.

### First-Order Correlations

**Fig. 6:**
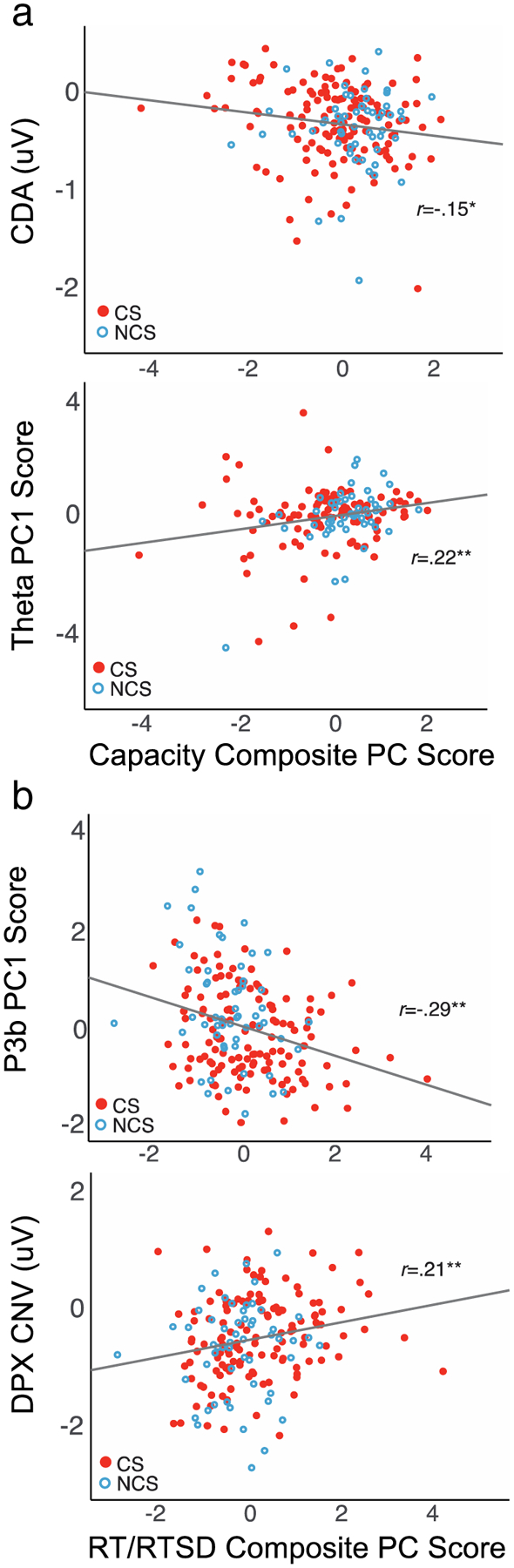
Scatterplots for first order correlations between composite behavioral outcome scores and EEG predictor variables that showed significant regression coefficient effects in multiple regression analysis (Table 5). **a)** Correlations with capacity composite principal component (PC) scores. Greater capacity was associated with more negative CDA in LCD task, and also greater theta power during maintenance, across all tasks. **b)** Correlations with RT/RTSD composite PC scores. Faster and less variable responses were associated with larger P3b amplitude during stimulus processing, across tasks, and more negative CNV in the 100 ms preceding X-stimuli in the DPX task. *p < 0.05; **p < 0.01

Finally, to further support interpretation of the above relationships, we evaluated the first-order correlations between composite outcome measures and the seven EEG predictors. As shown in Table 6 and Figure 6, first-order correlations were significant for significant predictors of composite capacity (CDA, theta) and composite RT/SDRT (P3b, CNV). Correlations among predictors were scarce with the exception of occipital alpha, which correlated negatively with P3b amplitude (*r(187)* = −0.24, *p* = 0.001), and with maintenance theta (*r(153)* = 0.17, *p* = 0.04), suggesting that this measure may play an additional indirect role in WM processes.

## Discussion

In this study we sought to test the relative contribution of putative WM processes, as measured by EEG features across four WM tasks, to WM performance. We found that storage sub-processes during maintenance (theta, CDA) were related to WM capacity, and were distinct from updating (P3b) and goal maintenance (CNV) processes, which were more strongly associated with reaction time measures. The results validate these putative subprocesses of WM and suggest that the most salient distinction in WM mechanisms lies in tonic processes during maintenance, versus phasic processes associated with stimulus processing. In addition, the findings highlight variations in the ability of WM tasks to measure different subprocesses and differences in reliability and validity of EEG measures, ranging from high (e.g., P3b, alpha ERD) to low (CFC, gamma).

### Subprocesses of WM

One contribution of the present work is in validating core subprocesses of WM, the identity and separability of which have been the subject of debate. For instance, the NIH RDoC framework (Insel et al., 2010) has proposed four putative domains of WM that include active maintenance, limited capacity, flexible updating, and interference control. Our results partially validate these dimensions and additionally suggest that active maintenance and limited capacity are coupled through their relationship to behavioral WM capacity measures, whereas updating and goal maintenance processes together are more associated with response speed and variability. The association of updating processes and response variability is notable, due to the potential role of such processes in contributing to WM impairments in ADHD (Lenartowicz et al., 2018; Lenartowicz et al., 2019). A caveat to our results, however, is that the chosen tasks were not designed to isolate WM updating processes from concurrent operations, such as stimulus categorization or automatic updating (Kessler, 2017; Rac-Lubashevsky & Kessler, 2016), and novel measures may be needed to provide greater sensitivity to these distinctions. Given that, in the service of dimension reduction, we collapsed across encoding and retrieval operations, it is likely that our results reflect general aspects of WM updating that involve interactions between sensory and associative systems.

In addition, some have debated whether the distinction between short-term storage (STM) and long-term storage (LTM) systems in WM is valid (Ranganath & Blumenfeld, 2005; Ruchkin, Grafman, Cameron, & Berndt, 2003), with several lines of research examining whether necessary conditions exist for the recruitment of cortico-hippocampal LTM mechanisms during WM maintenance (Axmacher et al., 2007; Rissman et al., 2008; Sakai & Passingham, 2004). Our results suggest that, across four tasks of variable demands, both LTM (theta) and STM (CDA) mechanisms are recruited during maintenance and both contribute to capacity, which broadly supports the view that minimizes distinctions between these systems in WM maintenance.

The results of this study may additionally support a simpler two-dimensional view of WM, namely the interplay between tonic processes that seek to maintain the stability of activated states free from interference from exogenous stimuli, and phasic processes that lead to shifting of activation states in response to novel stimuli and updating of response processing (Aston-Jones & Cohen, 2005; Bilder, 2012; Bilder, Volavka, Lachman, & Grace, 2004; Pribram & McGuinness, 1975). One of the identified behavioral dimensions coupled two maintenance-related features (frontal theta and CDA) and performance capacity scores, consistent with tonic processes. In contrast, the second dimension coupled P3b and CNV with reaction time metrics. The P3b is a putative correlate of WM updating (Polich, 2007) and, in alternate formulations, of categorization of content within WM (Kok, 2001; Nieuwenhuis et al., 2005). Thus, the P3b, such as the RT/RTSD measures, is consistent with phasic processes, during which sensory inputs trigger neural interactions and phasic reorganization of activity in the service of some outcome, like a response. The association of CNV with this dimension may appear contradictory, given that CNV was introduced as a correlate of goal maintenance. However, the CNV as we recorded it, is actually an ERP thought to index a combination of goal maintenance processes, and also, response preparation processes (Babiloni et al., 2005; Hamano et al., 1997). Thus, like the P3b, it is an indicator of phasic neurophysiological responses. From this perspective, the analysis casts WM as a function of tonic and phasic interactions that drive capacity and response outcomes, respectively.

Exploratory analyses showed that the behavioral assays of WM and the EEG measures acquired during WM tasks were related to clinical state variables in predicted ways; indeed, both the cognitive task measures and factors, and the EEG measures all differed between care-seeking (CS) and non-care-seeking (NCS) groups, with effect sizes in the “medium” range. We also noted modest correlations of both behavioral and EEG indicators with individual clinical variables. The P3b “updating” EEG measures appeared to have a broader impact across measures of clinical symptoms spanning depression and anxiety, while the CDA measure had more circumscribed associations with disability. While our study was not designed to address the hypothesis that there may be distinctive patterns of association between clinical symptoms and these WM indicators, future research might target these distinctions.

Finally, we highlight that a limitation of the study is a lack of non-WM control tasks. The absence of these control tasks limits interpretation of, in particular, the RT/RTSD factor, which may be a generic speed-related factor, not WM-related. Indeed, the dissociation of RT/RTSD from other accuracy-based indices has been reported previously in the context of clinical assessments in ADHD (Frazier-Wood et al., 2012; Kofler et al., 2014; Michelini et al., 2018). The present results expand on these observations by tying RT/RTSD with phasic neural responses, here measured by P3b and CNV. Similarly, the interpretation of our results is limited to the domain of spatial working memory, as verbal stimuli were excluded to control for individual differences in semantic knowledge. Additionally, we note that while certain task conditions are particularly important in assessing certain sub-processes (e.g., the DPX task was designed specifically to assess goal maintenance), it should be acknowledged that all of the tasks require all processes to some extent in order to succeed.

### EEG Metrics of WM

This study also highlights variability in reliability of EEG features previously associated with WM subprocesses. The P3b and alpha ERD both showed strong across-task reliability, comparable to that of WM performance across tasks, and replicated known load effects and group effects. The latter also was the case for CDA reported in the LCD task, and CNV reported in CPT paradigms. In contrast, gamma and theta showed weaker reliability (*Cronbach’s alpha* ~ 0.5), whereas CFC measures were unreliable (*Cronbach’s alpha* = 0.13), and across all these measures load and group effects failed to replicate. The underperformance of the CFC is likely coupled with weak reliability of gamma, reflected in weak across-task *Cronbach’s alpha*, an absence of load effects and, relatedly, non-parsimonious result in PCA on these metrics. Assessment of gamma at the scalp is notoriously difficult (Fries, Scheeringa, & Oostenveld, 2008; Lopes da Silva, 2013; Muthukumaraswamy, 2013) due to strong attenuation of high-frequency amplitudes by the scalp, as well as susceptibility of gamma-range frequencies to muscle generated artifacts (e.g., micro-saccades) (Yuval-Greenberg & Deouell, 2009; Yuval-Greenberg, Tomer, Keren, Nelken, & Deouell, 2008). Indeed, some of the most robust reports of gramma-range CFC findings were reported from intracortical recordings (Canolty et al., 2006; van Vugt, Schulze-Bonhage, Litt, Brandt, & Kahana, 2010; Voytek et al., 2010). Relatedly, prior reports of gamma modulation in scalp EEG recordings perhaps benefited from larger trial counts (e.g., n = 150, Roux & Uhlhaas, 2014; Roux, Wibral, Mohr, Singer, & Uhlhaas, 2012), than employed in the current study (n = 20–40 per condition). We conclude therefore that gamma range oscillations were not reliable in the current dataset due to limitations of EEG in measuring high frequencies and low power, not that these measures are poor indices of local cortical processing.

In contrast to gamma, theta-range power, a proposed index of long-term storage and/or cortico-hippocampal interactions, while also failing to show strong across-task reliability (*Cronbach’s alpha* = 0.5), did produce a parsimonious single component in principal component reduction and did show load effects, albeit inconsistently so. Theta power was also predictive of WM capacity, consistent with predictions. The results are therefore suggestive but lacking convergence. One possibility is that the chosen WM tasks did not generate the putative conditions (e.g., novelty, relational encoding, disruption of rehearsal, supra-capacity loads) posited as necessary for the recruitment of cortico-hippocampal LTM mechanisms during WM maintenance (Axmacher et al., 2007; Rissman et al., 2008; Sakai & Passingham, 2004). In some support of this hypothesis, the only task to show elevated power in the low alpha, high theta band during maintenance was the DFR task (Figure 3), which also was one of the most difficult (with respect to accuracy) and the only task that required maintenance of multiple complex objects (e.g., faces vs. spatial features). One surprising finding was that alpha ERD, despite strong reliability across tasks, did not correlate with WM performance, in contrast to prior reports (Lenartowicz et al., 2018). However, a key difference in the present sample was the sparsity of attentional deficits, suggesting that alpha ERD, and its associated attentional processes during stimulus encoding or retrieval, make only indirect contributions to WM performance that may manifest in performance when deficits arise. It is notable that alpha ERD was the only measure in the current analysis that was also correlated with other predictors of WM performance (e.g., P3b, Theta; Table 6), consistent with this prediction.

### WM Task Differences

A final contribution of the present study, the results demonstrate how WM can tasks differ in relative recruitment of different WM subprocesses. While all four tasks showed group differences (CS vs. NCS) in RT/RTSD, differences in measures of capacity were more pronounced in DPX and LCD tasks, than in DFR and SWM tasks. These observations imply that, first, phasic, stimulus-related processes associated with RT/RTSD, are reliable across tasks, also consistent with high across-task reliability measures for P3b and alpha ERD. Second, demands on capacity may differ between DPX/LCD and DFR/SWM tasks. One difference between these pairs is the task pace (Figure 1), with the former being relatively fast paced (<2-sec maintenance) and the latter being relatively slower paced (3-sec maintenance). Another difference is in type of encoding, with the latter being delay-match to sample tasks, and the former including change-detection (LCD) and matching to an internal template (DPX). Either of these differences could imply a greater sensitivity of DPX/LCD tasks to limited capacity or, in complement, a greater demand of SWM/DFR tasks on recruitment of complementary storage mechanisms, like corticohippocampal circuitry. As noted above, the DFR task is unique in being the only task to show elevated alpha/theta power during maintenance, along with lower accuracy, and increased complexity of to-be-stored objects. Consistent with this proposal, it is also the only task to show decreased P3b amplitude with load, previously suggested to arise with a change in encoding mechanisms with elevated task difficulty. In contrast, in SWM and LCD, P3b increased as load increased greater salience, a phenomenon consistent with prior observations of the P3b potential (Kok, 2001). Thus, we would hypothesize that DFR recruits LTM processes to a greater extent than spatial encoding (DPX, SWM) or dot-pattern recognition (DPX).

## Conclusions

The objective of this study was to test the relative contribution of putative WM processes, as measured by EEG features across four WM tasks, to WM performance. The results validate the contribution of short- and long-term maintenance processes (measured by CDA and theta) to WM capacity, in juxtaposition to the contribution of goal maintenance (CNV) and updating (P3b) to RT/RTSD. We suggest that the findings are consistent with a dual-process model of WM that sees variation in WM functions as the outcomes of balance between underlying tonic and phasic neurophysiological processes. Future studies will be needed to validate the strength of the relationship between the identified EEG metrics and underlying, putative circuitry of each WM process. The results additionally highlight that LCD and DPX tasks were more sensitive to capacity group differences, and therefore perhaps better suited to assessing related neural processes, and the DFR task perhaps better suited to long-term storage assessment. Finally, there exist clear differences in the across-task internal consistency of EEG features, with strongest consistency for P3b and alpha ERD (on par with reliability of performance across tasks), and negligible consistency for CFC metrics.

## Supplementary Material

Supplementary

## Figures and Tables

**Table 1:** Demographics

	CS	NCS	Group difference
Sample size	142	58	-
% Females	66.2%	56.9%	*x*^2^(1,200) = 1.5, *p* = 0.22
Mean age (SD)	28.4 (5.5)	27.1 (4.8)	t(198) = 1.6, *p* = 0.11
Years of education (SD)	17.3 (1.8)	17.2 (1.8)	t(198) < 1, *p* = 0.70
Estimated IQ (SD)	102.3 (11.6)	105.0 (12.4)	t(194) = 1.5, *p* = 0.30
**WHODAS 32-item Disability Score (SD)**	**20.6 (15.9)**	**6.12 (8.8)**	**t = 6.5, *p* < 0.0001**
*Annual household income*			
<$10,000	9%	3%	*x*^2^(5,200) = 2.9, *p* = 0.72
$10,000–$19,999	8%	2%	
$20,000–$39,999	15%	12%	
$40,000–$59,999	17%	14%	
$60,000–$99,999	15%	14%	
>$99,999	20%	17%	
**Unreported**	**16%**	**38%**	**X** ^**2**^ **(1,200) = 11.2, *p* = 0.001**
*Racial distribution*			
American Indian/Alaska Native	0.7%	1.7%	X^2^ (7,200) = 1.7, = 0.95
Asian	21.2%	24.1%	
Black or African American	9.2%	8.6%	
Hawaiian or Pacific Islander	2.8%	1.7%	
More than one race	9.2%	5.2%	
Unknown or unreported	11.3%	12.1%	
White	45.8%	46.6%	
Hispanic or Latino	31%	22.4%	***x***^2^(1,200) = 3.0, *p* = 0.23
Not Hispanic or Latino	66.9%	77.%	
*Primary Diagnosis*			
Mood	53.5%	19.0%	***x*** ^**2**^ **(6,200) = 59.8, *p* < 0.0001**
Anxiety	22.5%	10.3%	
Psychosis	5.6%	0.0%	
Eating disorder	0.7%	0.0%	
Anti-social personality disorder	0.7%	0.0%	
Attention deficit hyperactivity disorder	3.5%	3.4%	
No diagnosis	13.4%	67.2%	

*SD* = standard deviation; *WHODAS* = World Health Organization Disability Assessment Schedule 2.0; Significant differences (*p* < 0.05) indicated in bold

**Table 2:** Task performance

	CS Mean (SD)	NCS Mean (SD)	Group difference
*Delayed Face Recognition (DFR) Task Performance (n = 199)*			
K_DFR_	1.56 (0.53)	1.61 (0.42)	t < 1, *p* = 0.5
RT_DFR_	0.94 (0.17)	0.90 (0.13)	t = 1.85, *p* = 0.07
SDRT_DFR_	0.25 (0.06)	0.24 (0.06)	t = 1.68, *p* = 0.09
Accuracy_DFR_	0.84 (0.09)	0.86 (0.05)	t = 1.7, *p* = 0.09
#trials load1	41.4 (4.9)	41.3 (4.9)	t < 1, *p* = 0.93
#trials load3	32.8 (5.3)	33.1 (5.4)	t < 1, *p* = 0.72
*Sternberg Spatial Working Memory (SWM) Task Performance (n=199)*			
K_SWM_	5.19 (1.13)	5.48 (1.13)	t = 1.44, *p* = 0.15
**RT**_**SWM**_	**1.10 (.22)**	**0.96 (0.20)**	**t = 2.88, *p* = 0.004**
**SDRT**_**SWM**_	**0.34 (0.09)**	**0.32 (0.08)**	**t = 2.05, *p* = 0.04**
**Accuracy**_**SWM**_	**0.89 (0.08)**	**0.92 (0.05)**	**t = 2.6, *p* = 0.01**
#trials load1	20.6 (2.7)	20.8 (2.3)	t < 1, *p* = 0.57
#trials load3	19.5 (3.1)	20.3 (2.4)	t = 1.6, *p* = 0.11
#trials load5	18.6 (3.2)	19.0 (3.0)	t < 1, *p* = 0.45
#trials load7	18.4 (3.2)	19.0 (2.7)	t = 1.4, *p* = 0.19
*Lateralized Change Detection Task Performance (n = 196)*			
**K**_**LCD**_ (SD)	**2.53 (0.89)**	**2.81 (0.70)**	**t = 2.16, *p* = 0.03**
**RT**_**LCD**_ (SD)	**0.60 (0.15)**	**0.55 (0.10)**	**t = 2.27, *p* = 0.03**
**SDRT**_**LCD**_ (SD)	**0.23 (0.08)**	**0.19 (0.06)**	**t = 3.00, *p* = 0.003**
**Accuracy**_**LCD**_ **(SD)**	**0.82 (0.09)**	**.86 (.05)**	**t = 2.8, *p* = 0.005**
trials load1	26.4 (2.7)	26.7 (2.4)	t < 1, *p* = 0.38
**#trials load3**	**23.6 (3.5)**	**24.7 (3.0)**	**t = 2, *p* = 0.05**
#trials load5	20.2 (3.3)	20.9 (2.8)	t = 1.4, *p* = 0.15
*Dot-Pattern Expectancy Task Performance (n = 200)*			
**d’**_**DPX**_ (SD)	**3.00 (1.17)**	**3.37 (1.01)**	t **= 2.13, *p* = 0.03**
RT_DPX_ (SD)	0.44 (0.08)	0.43 (0.04)	t = 1.51, *p* = 0.13
**SDRT**_**DPX**_ (SD)	**0.12 (0.04)**	**0.11 (0.04)**	t = **2.52**, ***p* = 0.01**
Accuracy_DPX_ (SD)	0.94 (0.09)	0.96 (0.07)	t = 1.6, *p* = 0.11
#trials AX	134.8 (21.3)	136.1 (11.3)	t < 1, *p* = 0.67
#trials AY	20.9 (3.6)	20.9 (2.3)	t < 1, *p* = 0.97
#trials BX	21.8 (2.0)	21.6 (2.2)	t < 1, *p* = 0.58
#trials BY	10.6 (1.4)	10.6 (1.3)	t < 1, *p* = 0.93

SD = standard deviation; K = capacity score as defined in text; RT = median reaction time (seconds); SDRT = standard deviation of reaction time (seconds), d’ = d-prime, #trials = number of accurate trials in given condition. Significant differences (*p* < 0.05) indicated in bold

**Table 3:** Performance rotated component loadings

Measure	RT/RTSD composite component	Capacity composite component
K_DFR_	−0.025	**0.393**
K_SWM_	0.268	**0.828**
K_LCD_	0.063	**0.705**
d**’**DPX	−0.025	**0.763**
RT_DFR_	**0.737**	0.175
RT_SWM_	**0.699**	−0.184
RT_LCD_	**0.934**	*0.329*
RT_DPX_	**0.527**	−*0.308*
SDRT_DFR_	**0.644**	−0.009
SDRT_SWM_	**0.416**	−*0.303*
SDRT_LCD_	**0.767**	−0.049
SDRT_DPX_	**0.411**	−**0.576**

Loadings > 0.35 are in bold. Loadings > 0.3 are italicized to emphasize clustering of effects. Note that higher RT/RTSD composite scores indicate slower/more variable response time. Higher capacity composite scores indicate better capacity

**Table 4:** Working memory EEG features

Construct	Starting features	Tasks included				PCA	Cronbach’s *α* across tasks	Variable included in final analysis
Short Term Storage	Maintenance Gamma (frontal)*	DFR	SWM	LCD	DPX	✓	*0.46*	
	Maintenance Gamma (posterior)*	DFR	SWM	LCD	DPX		*0.50*	
	CDA			LCD			-	**CDA amplitude**
	N170	DFR					-	**N170 amplitude (O1 & O2)**
Long Term Storage	Maintenance Theta (frontal)	DFR	SWM	LCD	DPX	✓	0.52	**PC1: composite frontal theta**
	Maintenance Theta-Gamma CFC (frontal)*	DFR	SWM	LCD	DPX		*0.02*	-
	Maintenance Theta-Gamma CFC (posterior)*	DFR	SWM	LCD	DPX		*0.06*	-
Goal Maintenance & Control	Maintenance Alpha-Gamma CFC (frontal)*	DFR	SWM	LCD	DPX		*0.13*	-
	Maintenance Alpha-Gamma CFC (posterior)*	DFR	SWM	LCD	DPX		*0.02*	-
	CNV				DPX		-	**CNV amplitude (FCz)**
WM Updating	Stimulus P3b (Pz)	DFR	SWM	LCD	DPX	✓	0.72	**PC1: composite posterior P3b**
	Stimulus Alpha ERD (posterior)	DFR	SWM	LCD	DPX	✓	0.72	**PC1: composite posterior alpha ERD**
Alerting	P2 (frontal)		SWM				-	**P2 amplitude (Fz)**

*PCA* = principal component analysis; *PC* = principal component (scores);

* Excluded from final analysis due to low Cronbach’s Alpha across tasks

**Table 5:** Multiple regression: EEG predictors of working memory performance

Construct	Model predictors	DV: Capacity composite scores		DV: RT/RTSD composite scores	
		β	t_β_	β	t_β_
Short-term storage	**CDA amplitude**	−**0.21**	−2.72**	−0.07	0.86
	N170 amplitude (O1 & O2)	0.07	0.86	0.02	0.26
Long-term storage	**PC1: Frontal Theta**	**0.22**	**2.79** **	0.04	0.49
Goal maintenance	**CNV amplitude (FCz)**	−0.15	−1.91^†^	**0.16**	**2.02** *
WM updating	**PC1: composite posterior P3b**	0.16	1.92^†^	−**0.22**	−2.68**
	PC1: composite posterior alpha ERD	−0.05	0.60	0.10	1.23
Alerting	P2 amplitude (Fz)	0.03	0.40	0.06	0.76
	*Model Fit*	*R*^*2*^_*adj*_ = *0.17*, *F* = 2.91***, *n* = *153*		*R*^*2*^_*adj*_ = *0.10, F* = 2.64**, n = *153*	

† *p* < 0.1,

* *p* < 0.05,

** *p* < 0.01,

*** *p* < 0.001;

~ indicates that t_β_ < 1; DV = model dependent variable

**Table 6:** Variable correlations

	1	2	3	4	5	6	7	8	9
1. Capacity composite scores	*Performance*								
2. RT/RTSD composite scores	−0.41**								
3. CDA amplitude	−0.15*	−0.01	*STM*						
4. N170 amplitude (O1 & O2)	0.01	0.01	0.01						
5. PC1: Frontal Theta	**0.22** **	0.04	0.03	−0.06	*LTM*				
6. CNV amplitude (FCz)	−0.14	**0.21** **	0.06	0.12	−0.02	*GM*			
7. PC1: composite posterior P3b	**0.22** **	−0.29**	0	0.01	0	−0.07	*Updating*		
8. PC1: composite posterior alpha ERD	−0.06	0.08	0.02	−0.13	**0.17** *	−0.07	−0.24**		
9. P2 amplitude (Fz)	0.08	−0.05	−0.04	−0.08	−0.12	−0.12	0.03	−0.13	*Alerting*

STM = short-term storage, LTM = long-term storage, GM = goal maintenance.

† *p* < 0.1,

* *p* < 0.05,

** *p* < 0.01,

*** *p* < 0.001

## Open Practices Statement

The data and materials for all experiments are available upon request as well as on the NIMH National Data Archive (https://nda.nih.gov/). None of the experiments was preregistered.
