# Supplementary material for: Neurocognitive subprocesses of working memory performance
Source: Cogn Affect Behav Neurosci. 2021 Jun 21;21(6):1130–52. doi: 10.3758/s13415-021-00924-7 (PMC8563426; doi:10.3758/s13415-021-00924-7)
Supplement: Supplementary file 1 — (DOCX 38 kb) [file 13415_2021_924_MOESM1_ESM.docx]

**Supplemental Materials**

1. Diagnostic & Neurocognitive Assessment Details
2. ICA Methodological Details
3. Principal Component Within-Measure Dimension Reduction
4. Clinical Covariate Correlations

**1. Diagnostic & Neurocognitive Assessment Details**

Full results for the following measures will be presented in a separate publication and are omitted here due to a lack of significant associations between EEG indicators of WM and diagnostic quantifiers (Bilder et al., 2019)*.*

*Demographic, Developmental and Medical History:* The primary interview measure for both CS and NCS participants is the Composite International Diagnostic Interview (CIDI) developed by the World Health Organization (WHO). This measure has been extensively validated in populations around the world. The CIDI includes information on age, sex, education, marital status, employment, financial status, living arrangements, years in current country, primary ethnic identification, rural or urban upbringing, and parental SES and education level. Other data collected include social networks, previous psychiatric contacts and hospitalizations, medical conditions, and medication history. We used the WHO Disability Assessment Schedule (WHODAS-2.0; 36 items), which provides a well-validated global measure of disability and 6 domain-specific scales (Garin et al., 2010; Kessler & Ustun, 2004).

*Psychodiagnostic Measures*: The CIDI (Kessler & Ustun, 2004) was used to determine the presence, severity and disease burden of Axis I psychiatric disorders, including substance use. Interviews were administered by trained research staff satisfying reliability standards established by its designers. Substance use assessment included nicotine and alcohol use, plus age when substances were first used. Measures of symptoms were derived from the CIDI (syndromal severity and counts of symptoms in each module), and the DSM-5 crosscutting assessments (see below). To enable comparability to broader literature in psychopathology research we included the Brief Psychiatric Rating Scale (Thomas, Donnell, & Young, 2004; Ventura et al., 1993). Overall severity of psychological distress was indexed by a composite derived from the PROMIS-CAT, PQB (see above) along with the K-10 scale, which has been validated worldwide (Kessler et al., 2003; Kessler et al., 2010).

*Compatibility with DSM5:* The CIDI assesses disorders using the definitions and criteria of the Diagnostic and Statistical Manual of Mental Disorders, Fourth Edition (DSM-IV) and the ICD-10 Classification of Mental and Behavioral Disorders (ICD-10). To maximize forward compatibility with DSM-5 we also used the DSM-5-dimensional Level 1 and Level 2 measures of crosscutting symptoms (anxiety and mood symptoms, and general symptoms, including psychotic–like symptoms).

*Training and Reliability procedures:* CIDI training was obtained at a WHO-authorized CIDI Training and Reference Center. Training included home-study training materials (including 30 hours of home study) and 3 days of classroom training. Raters were required to pass certification procedures for each study instrument prior to conducting study assessments and satisfy on-site quality assurance procedures.

**2. ICA Methodological Details**

Individual-subject ICA results in many different ICs (sources) per subject, requiring comparable ICs from different subjects to be identified by some sort of similarity measure (c.f., Lenartowicz et al. (2014) for details). To do this, we used k-means clustering, implemented in EEGLAB, an iterative algorithm that assigns individual observations (ICs) from all subjects into *k* clusters, within a coordinate system defined by functional features (topography, event-related spectral change and voltage potential), such that each IC belongs to the cluster with the nearest centroid (Onton & Makeig, 2006). The algorithm would thus group, from all subjects, those ICs that have an occipital topography, for instance, into one cluster. It is possible, however, that some participants are excluded from a cluster, if their data did not produce an IC with the identifying cluster features, leading to small variation in sample size between features. The number of clusters was selected based on our prior analysis (Lenartowicz et al., 2014) and was set as 12. An additional, “outlier” cluster was identified for ICs whose distance to any cluster centroid was greater than 3 standard deviations. Clusters of interest were identified visually based on desired characteristics. Namely, we identified one cluster with mid-occipital topography and one cluster with mid-frontal topography to evaluate effects across known occipital and frontal scalp distributions.

**3. Principal Component Within-Measure Dimension Reduction**

The PCA loadings for each task in within-measure analyses are included below. Within-measure, across-task analyses were performed for measures that were available in all four tasks and that had a *Cronbach’s alpha* reliability index > .5: maintenance gamma, frontal maintenance theta, and also P3b and alpha ERD during stimulus processing.

As shown in Table S1, gamma features did not reveal a common underlying dimension across tasks. Four components were identified. Components 1 and 4 loaded primarily on frontal versus occipital gamma metrics (respectively) in the DPX and LCD tasks, component 2 loaded only on gamma in the SWM task, and component 3 loaded only on gamma in the DFR task. Thus, maintenance gamma did not show a common component across all WM tasks. In contrast, maintenance theta, P3b and alpha ERD produced a single PC loading across all four tasks (Tables S2-S4).

| **Table S1. *Maintenance Gamma Rotated Component Loadings*** | | |  |  |
| --- | --- | --- | --- | --- |
| **Measure** | **PC1** | **PC2** | **PC3** | **PC4** |
| DFR gamma frontal IC | -.07 | .04 | **.84** | .24 |
| DPX gamma frontal IC | **.41** | .09 | .14 | **.72** |
| LCD gamma frontal IC | -.08 | .13 | -.01 | **.87** |
| SCAP gamma frontal IC | -.11 | **.89** | .09 | .25 |
| DFR gamma occipital IC | .16 | .06 | **.85** | -.13 |
| DPX gamma occipital IC | **.89** | -.03 | .09 | .15 |
| LCD gamma occipital IC | **.86** | .01 | .00 | -.02 |
| SCAP gamma occipital IC | .10 | **.95** | .03 | -.01 |
| *Notes. Loadings > .35 are in bold. Loadings > .3 are italicized to emphasize clustering of effects.* | | | | |

| **Table S2. *Maintenance Theta Component Loadings*** | |  |
| --- | --- | --- |
| **Measure** | **PC1** | |
| DFR | **.79** | |
| DPX | **.70** | |
| LCD | **.77** | |
| SWM | *.33* | |
| *Notes. Loadings > .35 are in bold. Loadings > .3 are italicized to emphasize clustering of effects.* | |  |

| **Table S3. *Stimulus P3b Component Loadings*** | |  |
| --- | --- | --- |
| **Measure** | **PC1** | |
| DFR | **.70** | |
| DPX | **.87** | |
| LCD | **.83** | |
| SWM | *.***78** | |
| *Notes. Loadings > .35 are in bold. Loadings > .3 are italicized to emphasize clustering of effects.* | |  |

| **Table S4. *Stimulus Alpha ERD Component Loadings*** | |  |
| --- | --- | --- |
| **Measure** | **PC1** | |
| DFR | **.64** | |
| DPX | **.85** | |
| LCD | **.84** | |
| SWM | *.***81** | |
| *Notes. Loadings > .35 are in bold. Loadings > .3 are italicized to emphasize clustering of effects.* | |  |

**4. Clinical covariate correlations**

We examined the first-order correlations between EEG features used in multiple regression analyses and clinical covariate measures: WHODAS overall disability index, depression and anxiety subscale theta scores for the BRIEF scales, and depression and anxiety scores for the PROMIS scales. As shown in table S5 significant correlations were observed primarily for the P3b, and less reliably for CDA and CNV. All correlations were in the expected direction: EEG features weakened in magnitude with increasing scores on clinical indicators.

| **Table S5. *Correlations between clinical covariates and EEG indicators*** | | | | |  |  |
| --- | --- | --- | --- | --- | --- | --- |
|  | WHODAS | BPRS Anxiety | BPRS Depression | PROMIS Anxiety | | PROMIS Depression |
| 3. CDA amplitude | **.14*** | .12 | **.17*** | .05 | | .06 |
| 4. N170 amplitude (O1 & O2) | -.01 | -.07 | -.03 | .02 | | .04 |
| 5. PC1: Frontal Theta | -.13 | -.05 | -.11 | .03 | | -.05 |
| 6. CNV amplitude (FCz) | .06 | .01 | **.15*** | .02 | | .06 |
| 7. PC1: composite posterior P3b | **-.24**** | **-.23**** | **-.20**** | **-.20**** | | **-.18*** |
| 8. PC1: composite posterior alpha ERD | -.06 | .13 | -.02 | .04 | | -.02 |
| 9. P2 amplitude (Fz) | .01 | .01 | -.04 | -.02 | | -.01 |
| *Notes. *p<.05, **p<.01, ***p<.001* | | | | |  |  |

References

Bilder, R. M., Lenartowicz, A., Rissman, J., Loo, S., Pochon, J. B., Enriquez, K., . . . Hellemann, G. (2019). *Spanning levels in the RDoC Matrix: Does working memory work?* Paper presented at the Society for Biological Psychiatry, Chicago.

Garin, O., Ayuso-Mateos, J. L., Almansa, J., Nieto, M., Chatterji, S., Vilagut, G., . . . consortium, M. (2010). Validation of the "World Health Organization Disability Assessment Schedule, WHODAS-2" in patients with chronic diseases. *Health Qual Life Outcomes, 8*, 51. doi:10.1186/1477-7525-8-51

Kessler, R. C., Barker, P. R., Colpe, L. J., Epstein, J. F., Gfroerer, J. C., Hiripi, E., . . . Zaslavsky, A. M. (2003). Screening for serious mental illness in the general population. *Arch Gen Psychiatry, 60*(2), 184-189. doi:10.1001/archpsyc.60.2.184

Kessler, R. C., Green, J. G., Gruber, M. J., Sampson, N. A., Bromet, E., Cuitan, M., . . . Zaslavsky, A. M. (2010). Screening for serious mental illness in the general population with the K6 screening scale: results from the WHO World Mental Health (WMH) survey initiative. *Int J Methods Psychiatr Res, 19 Suppl 1*, 4-22. doi:10.1002/mpr.310

Kessler, R. C., & Ustun, T. B. (2004). The World Mental Health (WMH) Survey Initiative Version of the World Health Organization (WHO) Composite International Diagnostic Interview (CIDI). *Int J Methods Psychiatr Res, 13*(2), 93-121. doi:10.1002/mpr.168

Lenartowicz, A., Delorme, A., Walshaw, P. D., Cho, A. L., Bilder, R. M., McGough, J. J., . . . Loo, S. K. (2014). Electroencephalography correlates of spatial working memory deficits in attention-deficit/hyperactivity disorder: vigilance, encoding, and maintenance. *J Neurosci, 34*(4), 1171-1182. doi:10.1523/JNEUROSCI.1765-13.2014

Onton, J., & Makeig, S. (2006). Information-based modeling of event-related brain dynamics. *Event-Related Dynamics of Brain Oscillations, 159*, 99-120. doi:Doi 10.1016/S0079-6123(06)59007-7

Thomas, A., Donnell, A. J., & Young, T. R. (2004). Factor structure and differential validity of the expanded Brief Psychiatric Rating Scale. *Assessment, 11*(2), 177-187. doi:10.1177/1073191103262893

Ventura, J., Lukoff, D., Nuechterlein, K., Liberman, R., Green, M., & Shaner, A. (1993). *Brief Psychiatric Rating Scale (Expanded Version 4.0)*. Retrieved from
